# DNA methylation signal has a major role in the response of human breast cancer cells to the microenvironment

**DOI:** 10.1038/oncsis.2017.88

**Published:** 2017-10-23

**Authors:** P Mathot, M Grandin, G Devailly, F Souaze, V Cahais, S Moran, M Campone, Z Herceg, M Esteller, P Juin, P Mehlen, R Dante

**Affiliations:** 1 Dependence Receptors, Cancer and Development Laboratory, Centre de Recherche en Cancérologie de Lyon (CRCL), Inserm U1052-CNRS UMR5286, Université de Lyon, Centre Léon Bérard, Lyon, France; 2 Department of Developmental Biology, The Roslin Institute, University of Edinburgh, Easter Bush Campus, Midlothian, UK; 3 Cell survival and tumor escape in breast cancer Laboratory, Center for Cancer Research Nantes-Angers UMR 892 Inserm—6299 CNRS/Université de Nantes, Nantes, France; 4 Epigenetics Group, IARC, Lyon, France; 5 Cancer Epigenetics and Biology Program (PEBC), Bellvitge Biomedical Research Institute (IDIBELL), L’Hospitalet, Barcelona, Spain; 6 Physiological Sciences Department, School of Medicine and Health Sciences, University of Barcelona (UB), Barcelona, Spain; 7 Institucio Catalana de Recerca i Estudis Avançats (ICREA), Barcelona, Spain

## Abstract

Breast cancer-associated fibroblasts (CAFs) have a crucial role in tumor initiation, metastasis and therapeutic resistance by secreting various growth factors, cytokines, protease and extracellular matrix components. Soluble factors secreted by CAFs are involved in many pathways including inflammation, metabolism, proliferation and epigenetic modulation, suggesting that CAF-dependent reprograming of cancer cells affects a large set of genes. This paracrine signaling has an important role in tumor progression, thus deciphering some of these processes could lead to relevant discoveries with subsequent clinical implications. Here, we investigated the mechanisms underlying the changes in gene expression patterns associated with the cross-talk between breast cancer cells and the stroma. From RNAseq data obtained from breast cancer cell lines grown in presence of CAF-secreted factors, we identified 372 upregulated genes, exhibiting an expression level positively correlated with the stromal content of breast cancer specimens. Furthermore, we observed that gene expression changes were not mediated through significant DNA methylation changes. Nevertheless, CAF-secreted factors but also stromal content of the tumors remarkably activated specific genes characterized by a DNA methylation pattern: hypermethylation at transcription start site and shore regions. Experimental approaches (inhibition of DNA methylation, knockdown of methyl-CpG-binding domain protein 2 and chromatin immunoprecipitation assays) indicated that this set of genes was epigenetically controlled. These data elucidate the importance of epigenetics marks in the cancer cell reprogramming induced by stromal cell and indicated that the interpreters of the DNA methylation signal have a major role in the response of the cancer cells to the microenvironment.

## Introduction

The tumor microenvironment is composed of a heterogeneous population of non-neoplastic cells including immune cells, vascular endothelial cells and fibroblasts. The presence of cancer cells leads to the appearance of phenotypically modified fibroblasts, which in turn reprogram tumor cells. The cross-talk between these components and cancer cells promotes tumor growth, metastasis,^1^ and impacts the response of tumors to anti-cancer therapies.^2, 3, 4, 5, 6, 7, 8^

In breast tumors-activated fibroblasts (cancer-associated fibroblasts, CAFs) are the predominant stromal cell type.^9^ CAFs present some characteristics of myofibroblasts and express specific markers including α-smooth muscle actin, vimentin, neuron glial antigen-2 and fibroblast-specific protein-1.^10^ CAFs secrete various growth factors (including, fibroblast growth factors, hepatocyte growth factor, transforming growth factor-β1 and SDF-1/CXCL12), cytokines (including, IL-6, Slit2, IL-8, IL-10, TNF-α, monocyte chemoattractantprotein-1, leptin and interferon-β), proteases and extracellular matrix components involved in tumor initiation, progression and invasion of breast cancer.^11, 12, 13, 14, 15, 16, 17, 18^ *In vitro* co-cultures have underlined the importance of heterotypic interactions among different cell types.^19^ For instance, the contribution of CAFs to lapatinib resistance can be mediated by cell contact,^7^ whereas CAF culture supernatants are able to induce resistance to RAF inhibitors in breast cancer cells.^16, 20^ Soluble factors secreted by CAFs and cancer cells are involved in many pathways including inflammation, metabolism, proliferation and epigenetic modulation,^21^ suggesting that CAF-dependent reprograming of cancer cells affects a large set of genes.

Thus, paracrine signaling seems to have an important role in the cross-talk between cancer cells and CAFs, and deciphering some of these processes could lead to relevant discoveries with subsequent clinical implications. In the present study, we have investigated the mechanisms underlying the changes in gene expression patterns associated with the cross-talk between cancer cells and the stroma. Unexpectedly, we found that gene expression changes induced by CAF-secreted factors were not mediated through significant DNA methylation changes. Nevertheless, CAF-secreted factors remarkably activate genes characterized by a high level of methylated CpGs on their regulatory region, defining a DNA methylation pattern of genes modulated by stromal cell contents in human breast tumors. Our data put in light the importance of epigenetic marks in the cancer cell reprogramming induced by stromal cell.

## Results

### Identification of stromal-dependent genes in human breast tumors

**Figure 1:**
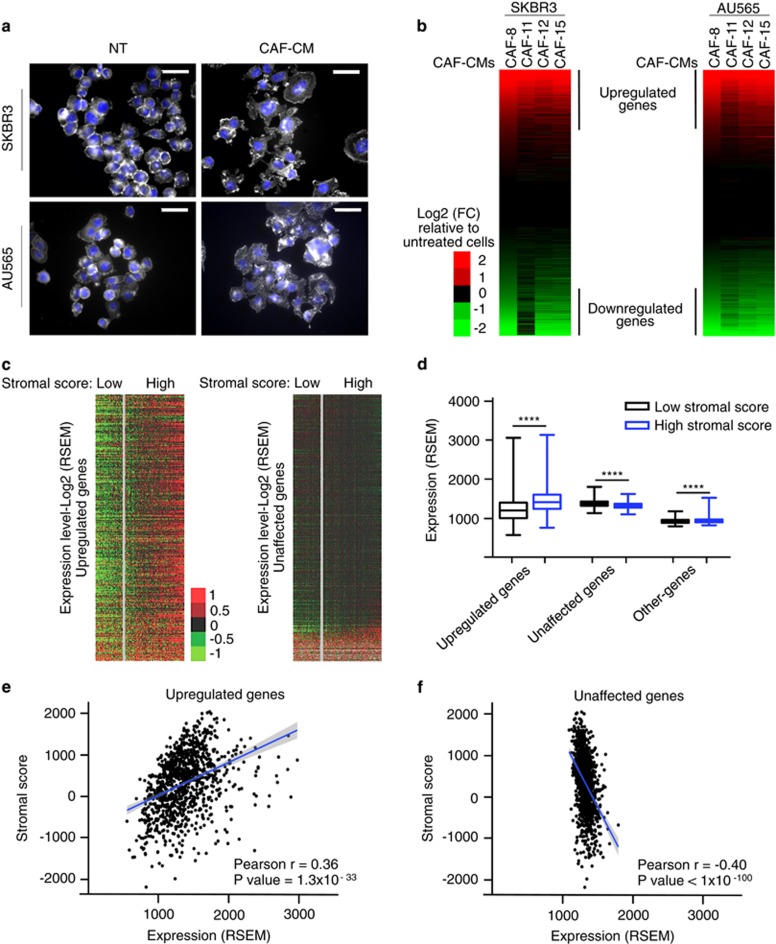
Identification of stromal-dependent genes in human breast tumors. (**a**) Breast cancer cell lines SKBR3 and AU565 treated with CAF-11 CM or control medium for 48 h were stained for F-actin. Nuclei were visualized using DAPI staining in blue. Representative images, scale bars, 50 μm. (**b**) Heatmaps from RNAseq data of SKBR3 and AU565 cell lines. Genes were classified according to their expression levels in cells treated with CAF-CMs normalized by their expression in control cells. (**c**) Heatmap depicting expression level of genes modulated by CAF-secreted factors identified *in vitro* in function of human breast tumors stromal score; left panel: genes upregulated by CAF-secreted factors (median centering, *n*=1042); right panel: genes unaffected by CAF-secreted factors (median centering, *n*=1042). (**d**) Mean expression level of genes modulated by CAF-secreted factors identified *in vitro,* in human breast tumors as a function of the tumor stromal score. (****P<0.0001, two-tailed unpaired Student’s t-test. Error bars=s.e.m). (**e**) Linear regression curve presenting the relationship between the mean expression level of genes upregulated by CAF-secreted factors identified *in vitro* and the tumor stromal score. (**f**) Linear regression curve presenting the relationship between genes unaffected by CAF-secreted factors identified *in vitro* and the tumor stromal score.

To assess the mechanisms underlying the changes in gene expression patterns associated with the cross-talk between cancer cells and the stroma, primary cultures of stromal fibroblasts were established from three primary infiltrating ductal carcinoma (CAF-8, CAF-11 and CAF-15) and from one primary infiltrating lobular carcinoma (CAF-12); the anatomopathological characteristics of the breast tumors are shown in Supplementary Table S1. Western blot analysis of the cultured fibroblasts (CAF-8, CAF-11, CAF-12 and CAF-15) indicated that they strongly expressed the CAF markers, alpha smooth muscle actin and vimentin,^10^ at a similar level at different passages, whereas, as expected, the negative marker E-cadherin remained undetected (Supplementary Figure S1). CAF cultures were also characterized by their impact on cancer cell morphology. When cultured for 48 h in CAF culture supernatant (CAF-CM), the SKBR3 and the AU565 breast cancer cell lines underwent morphological changes, adopting a spindle-like shape with actin reorganization and an increase in size (Figure 1a), consistent with other breast cancer cell lines cultured in CAF culture supernatants^22^ or treated with transforming growth factor-β.^23^

To identify gene expression changes induced by CAF-secreted factors, the impact of CAF-CM on the transcriptomes of SKBR3 and AU565 breast cancer cell lines was investigated by high-throughput sequencing of polyadenylated RNA (RNAseq). Heatmaps depicting fold changes in gene expression induced by CAF-CMs indicated that conditioned media from the four primary cultures of CAF have similar effects on gene expression in both cell lines (Figure 1b). Combining data obtained using CAF-8, CAF-11, CAF-12 and CAF-15, we identified 558 and 732 genes upregulated (fold change (FC)⩾2, *q*-value<0.05), respectively, in SKBR3 and AU565 cells exposed to CAF-CMs (Supplementary Figure S2). Furthermore, the fold changes induced by CAF-CMs were highly correlated between each other (Pearson correlation coefficient, *r*, ranging from 0.902 to 1, Supplementary Table S2).

Similarities between gene induction by CAF were also observed between cell lines, as 67% of genes upregulated upon exposure to CAF-CMs in SKBR3 cells were also upregulated in AU565 (Supplementary Figure S3A). These data led to the identification of a set of 372 genes upregulated (FC⩾2, in both cell lines) by CAF-secreted factors in both cell lines (‘upregulated genes’ Supplementary Table S5). Concomitantly we identified a set of 3479 genes unaffected by CAF-secreted factors (0.8⩽FC⩽1.2, in both cell lines), in SKBR3 and AU565 cell lines (‘unaffected genes’ Supplementary Table S5). ‘Upregulated genes’ were investigated for their representation in KEGG pathways by WebGestalt.^24^ KEGG pathways enriched in this set of genes included several pathways known to be involved in the cross-talk between CAFs and cancer cells, metabolic pathways, cytokine–cytokine receptor interaction, pathways implicated in cancer, the Jak-STAT signaling pathway, and the MAPK signaling pathway (Supplementary Figure S3B).

These *in vitro* results identified groups of genes modulated by CAF-secreted factors in breast cancer cell lines. In order to investigate the potential physiological significance of this finding, we examined their expression according to the level of stromal cells present in human breast tumors from samples available through The Cancer Genome Atlas (TCGA). The stromal cell contents of the tumors were evaluated using the stromal ESTIMATE score,^25^ (data available at bioinformatics.mdanderson.org/estimate). Among the 372 ‘upregulated genes’, only 6 also belonged to the gene list of stromal signature.^25^ Tumors were subdivided in two groups, ‘high stromal score’ (positive score values, *n*=750) and ‘low stromal score’ (negative score values, *n*=350). Heatmap depicting the expression level of the set of ‘upregulated genes’ in the two groups of tumors (high/low stromal score) indicated that the set of ‘upregulated genes’ was expressed at a higher level in the group of tumors with high stromal cell content than in the group with low stromal cell content (Figure 1c). Conversely, the expression levels of the ‘unaffected genes’ were similar in the two groups of tumors (Figure 1c).

Then, these differences were quantified using the mean expression levels of the different groups of genes: ‘upregulated genes’, ‘unaffected genes’ and ‘other-genes group’ (genes not included in the two later categories) (Figure 1d). For the set of ‘upregulated genes’, the difference between means observed between the two groups of tumors was statistically significant (two-tailed unpaired Student’s *t*-test, *P*<0.0001, difference between means in RSEM (diff-mean-RSEM)=218.2, coefficient of variation (CV=24.9%). In contrast, very small differences were observed between the two groups of tumors for the ‘unaffected genes’ (diff-mean-RSEM=–57.50, CV=6.6%) and for the ‘other-genes group’ (diff-mean-RSEM=17.65, CV=7.2%) (Figure 1d). Furthermore, the mean expression level (ranging from 563 to 3125 RSEM) of the ‘upregulated genes’ was positively correlated with the stromal score (Pearson’s *r*=0.36, *P*=1.3 × 10^−33^) (Figure 1e). This positive correlation was not observed for the ‘unaffected genes’, their mean expression level varied in a narrow range (from 1093 to 1793 RSEM) and was even negatively correlated (Pearson’s *r*=−0.40, *P*<1 × 10^−100^) with the stromal score (Figure 1f).

When the human breast tumors were subdivided according to the main tumor types found in the TCGA databank (invasive lobular carcinoma and invasive ductal carcinoma samples), this correlation seemed to be stronger (Pearson’s *r*=0.49) in invasive lobular carcinoma than in invasive ductal carcinoma (Pearson’s *r*=0.33) (Supplementary Figure S4). Although this difference may be due to sample sizes (*n*=182 and *n*=752), these data may suggested that the response to stromal cell content might be also dependent on the tumor type. Taken together, these results identified groups of genes, which are modulated by CAF-secreted factors *in vitro* and presenting an expression level dependent on the presence of stromal cells in human breast tumors.

### Gene upregulation upon CAF-secreted factors occurs in the absence of DNA methylation changes

**Figure 2:**
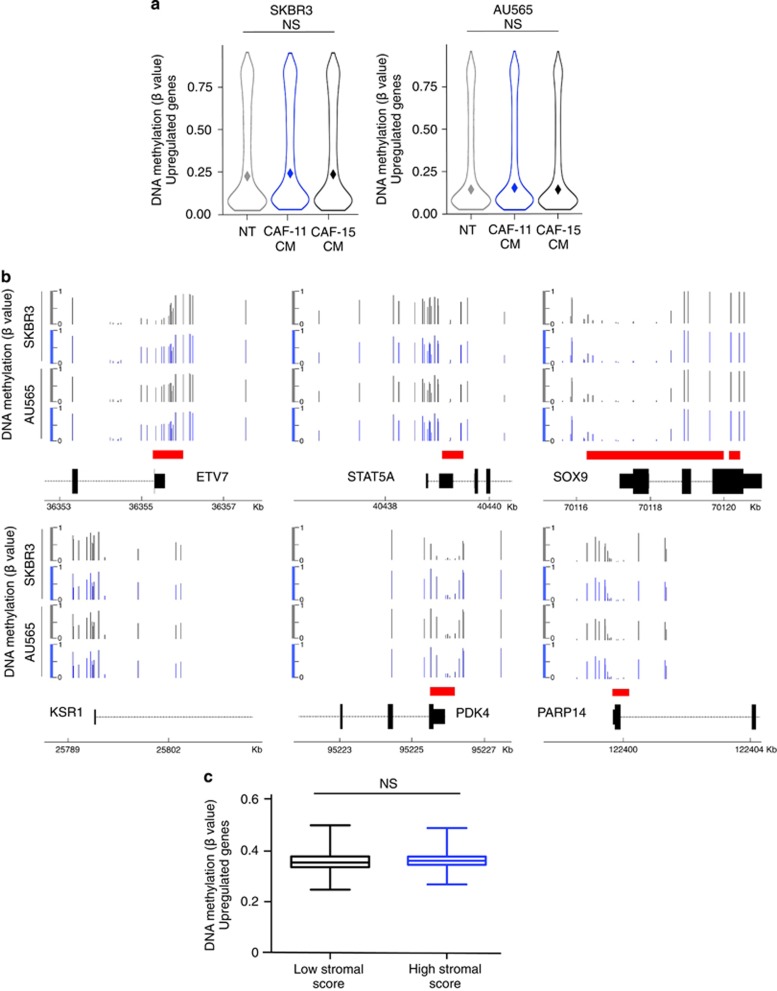
Gene upregulation upon CAF-secreted factors occurs in the absence of DNA methylation changes. (**a**) Violin plots depicting the level of DNA methylation (β value) at TSS1500 regions of ‘upregulated genes’. The level of DNA methylation of these genes was analyzed in SKBR3 and AU565 cells treated with CAF-11 CM, CAF-15 CM or control medium (NT) for 48 h. (**b**) Genome coverage of DNA methylation in NT cells (gray) or in cells treated with CAF-11 CM (blue) at six genomic locations. Scales (β values); Genes in black and CpG islands in red. (**c**) Mean DNA methylation level (β values) at TSS1500 regions of ‘upregulated genes’ in human breast tumors in function of their tumor stromal score. (NS *P*⩾0.05, two-tailed unpaired Student’s *t*-test).

Epigenetic modifications seem to have a crucial role in the cross-talk between cancer cells and the stroma.^26, 27^ To investigate the existence of DNA methylation changes associated with genes modulation induced by CAF-secreted factors, DNA methylation levels, at key genomic regions, were determined using the MethylationEPIC (EPIC) BeadChip technology, which covers over 850 000 CpG sites. We observed first that DNA methylations levels, at key genomic regions, were different in SKBR3 and AU565 cells (Supplementary Figure S5). CpGs were annotated according to the manifest provided with the Infinium MethylationEPIC and classified into three different groups: ‘TSS regions’ (TSS1500, 0–1500 bases upstream of transcription start site (TSS), ‘shore regions’ (shore, 0–2 kb from CpG island) and ‘shelf regions’ (shelf, 2–4 kb from CpG island). Differential methylation was not observed on the TSS regions of the ‘upregulated genes’ in SKBR3 and AU565 cells grown in two different CAF-CMs (Figure 2a). Data obtained for *ETV7*, *STAT5A*, *SOX9*, *KSR1*, *PDK4* and *PARP14* illustrate this analysis (Figure 2b). Furthermore, using ChAMP software, we did not detect significant change at the 850 000 CpGs analyzed, in cell lines grown in CAF-CMs compared with control cells. Concomitantly, in human breast tumor we did not detect differential methylation of ‘upregulated genes’ in tumor presenting high stromal score compare with tumor presenting low stromal score (Figure 2c). Although we,^28, 29, 30^ as many other colleagues, have observed that methylation changes may have a major role in carcinogenesis, our data showed that genes induced by stromal cells was unexpectedly not mediated through DNA methylation changes.

### DNA methylation patterns of genes modulated by CAF-secreted factors

**Figure 3:**
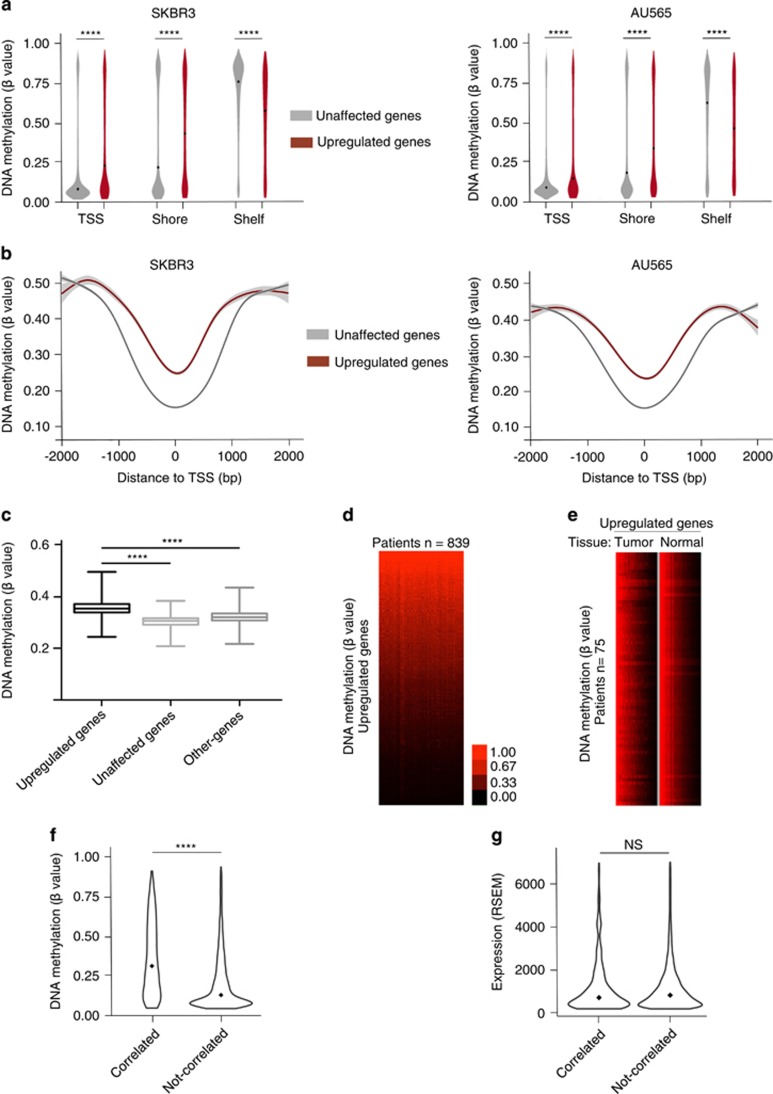
DNA methylation of genes modulated by CAF-secreted factors. (**a**) Violin plots depicting the level of DNA methylation (β values) of ‘upregulated genes’ or ‘unaffected genes’ in SKBR3 and AU565 cell lines; diamond, median value; DNA methylation level at TSS1500, Shore and Self-regions. (****P<0.0001, two-tailed unpaired Student’s *t*-test). (**b**) DNA methylation level (β values) of ‘upregulated genes’ or ‘unaffected genes according to their distance to TSS, in SKBR3 and AU565 cell lines. (**c**) Mean DNA methylation levels at the TSS1500 region of genes modulated by CAF-secreted factors identified *in vitro*, in human breast tumors. (****P<0.0001, two-tailed unpaired Student’s *t*-test). (**d**) Heatmap depicting the methylation level at TSS1500 regions of ‘upregulated genes’, in human breast tumors (*n*=839 patients). (**e**) Heatmap depicting the methylation level at TSS1500 regions of ‘upregulated genes’ in paired normal/tumoral sample, in the 75 patients analyzed. (**f**) Violin plots depicting the level of DNA methylation (β values) of genes with an expression positively correlated (‘Correlated’, Pearson *r*⩾0.3, *n*=736) or not correlated (‘Not-correlated’, Pearson *r*<0.3, *n*=8647) with the stromal cell content of the tumors; diamond, median value; DNA methylation level at TSS1500 (****P<0.0001, two-tailed unpaired Student’s *t*-test). (**g**) Violin plots depicting the expression level of genes Correlated or Not-correlated with the stromal cell content of the tumors, diamond, median value; Expression level (NS=not significant, *P*=0.75, two-tailed unpaired Student’s *t*-test).

We observed that the TSS regions of ‘upregulated genes’ were significantly more methylated (*P*<0.0001, two-tailed unpaired Student’s *t*-test) than the TSS regions of ‘unaffected genes’, in SKBR3 and AU565 cells (Figure 3a). Significant hypermethylation of ‘upregulated genes’ (*P*<0.0001, two-tailed unpaired Student’s *t*-test) was also observed for shore regions (Figure 3a), whereas shelf regions that are more distant from regulatory regions did not exhibited this characteristic (Figure 3a). This hypermethylation was symmetrically distributed around the TSS for both cell lines (Figure 3b). Furthermore, in breast tumor, as observed in cell lines, the set of ‘upregulated genes’ exhibited hypermethylations on their TSS regions when compared with the TSS region of ‘unaffected genes’ or the TSS region of the ‘other-genes groups’ (two-tailed unpaired Student’s *t*-test, *P*<0.0001) (Figure 3c). Heatmap depicting the TSS methylation level of ‘upregulated genes’ on human breast tumors revealed a relative homogeneity among the 839 patients analyzed (Figure 3d). Moreover, heatmap depicting the mean methylation level of these ‘upregulated genes’ in tumor tissue and normal tissue in paired normal/tumoral samples (*n*=75) showed similar DNA methylation levels of these genes in tumor when compared with their normal counterpart, indicating that this DNA methylation pattern was not induced by the tumor but, for most of them, characterized these genes (Figure 3e).

Then, as a control, we investigated the relationship between genes harboring an expression level positively correlated with the stromal cell content of the tumors and their methylation statue at TSS regions, from the TCGA database. TGCA breast cancer cohort was subdivided in genes correlated (Pearson *r*⩾0.3), or not correlated (Pearson *r*<0.3), with the stromal cell content of the tumors. We observed that DNA methylation levels at TSS regions were higher in the group of correlated genes (% of methylation: median value: 0.35; *N*=1066 genes) than in the group of genes not-correlated (median value: 0.21; *N*=14904). To avoid potential biases due to different expression levels between the two groups, we also limited our analysis to genes with similar expression levels (2 × median⩽RSEM values⩾0.5 × median). Differential methylation levels between the two groups were still observed (median value: 0.30, *N*=736 versus median value: 0.10, *N*=8647 in the group genes correlated or not correlated, respectively) (Figure 3f), whereas their mean expression level was not significantly different (two-tailed unpaired Student’s *t*-test, *P*=0.75), (Figure 3g).

This result indicate that CAF-secreted factors but also stromal content of the tumor remarkably activate genes characterized by a high level of methylated CpGs on their regulatory region, defining a DNA methylation pattern of genes modulated by stromal cell contents in human breast tumors. Thus, this DNA methylation pattern of genes upregulated by CAF-secreted factors suggests that DNA methylation was involved in their transcriptional regulation.

### Involvement of DNA methylation in the response to CAF-secreted factors

**Figure 4:**
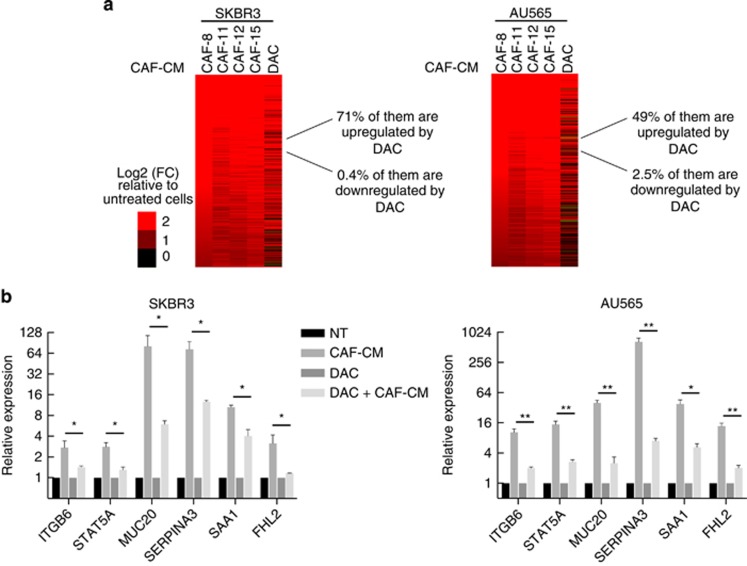
Involvement of DNA methylation in the response to CAF-secreted factors. (**a**) Heatmaps depicting the set of ‘upregulated genes’ upon exposure to CAF-CMs or DAC. (**b**) Relative expression of six genes in SKBR3 and AU565 cells treated with CAF-11-CM (CAF-CM), decitabine (DAC) or both (DAC+CAF-CM) (*P<0.05; **P<0.01, two-tailed unpaired Student’s *t*-test. Error bars=s.e.m). NT=not treated.

To gain further insight into the association between these DNA methylation patterns and gene expression, SKBR3 and AU565 cells were treated with decitabine (DAC), an inhibitor of DNA methylation. Analysis of CpG methylation using the EPIC BeadChip approach indicated that DAC treatment resulted in an efficient demethylation of TSS regions (*P*<0.0001, two-tailed unpaired Student’s *t*-test) as the global hypomethylation levels, at the CpG represented on these beads, were estimated at 18% and 27% for SKBR3 and AU565 cells, respectively (Supplementary Figure S6). Furthermore, RNAseq experiments, indicated that a large proportion (70% and 49%, in SKBR3 and AU565 cells, respectively) of genes upregulated by CAF-CMs were also upregulated upon DNA methylation inhibition by DAC (FC⩾2, adjusted *P*<0.05), whereas DAC treatments downregulated a very small fraction (0.4% and 2.5%, in SKBR3 and AU565 cells, respectively) of this set of genes (Figure 4a). Moreover, a very low proportion (0.9% and 1.3%, in SKBR3 and AU565 cells, respectively) of genes unaffected by CAF-CMs were upregulated upon DAC treatment (Supplementary Figure S7), suggesting that DNA methylation was involved in the regulation of a large part of ‘upregulated genes’.

The potential involvement of DNA methylation in the response to CAF-secreted factors was investigated for specific genes in both cell lines. For *ITGB6*, *STAT5A*, *MUC20*, *SERPINA3*, *SAA1* and *FHL2*, DAC treatment reduced their response to CAF-CM (Figure 4b). This result indicates that CAF-mediated gene upregulation and demethylation-dependent upregulation (induced by DAC) did not produce a cumulative effect. This result suggests that presence of methylated CpGs seems to have an important role on the upregulation induced by CAF-secreted factors. As the upregulation of these hypermethylated genes was not mediated through a demethylation induced by CAF-secreted factors, it might be dependent on the modulation of proteins involved in the interpretation of their DNA methylation.

### Methyl DNA-binding protein MBD2 deposition at genes modulated by CAF-secreted factors

**Figure 5:**
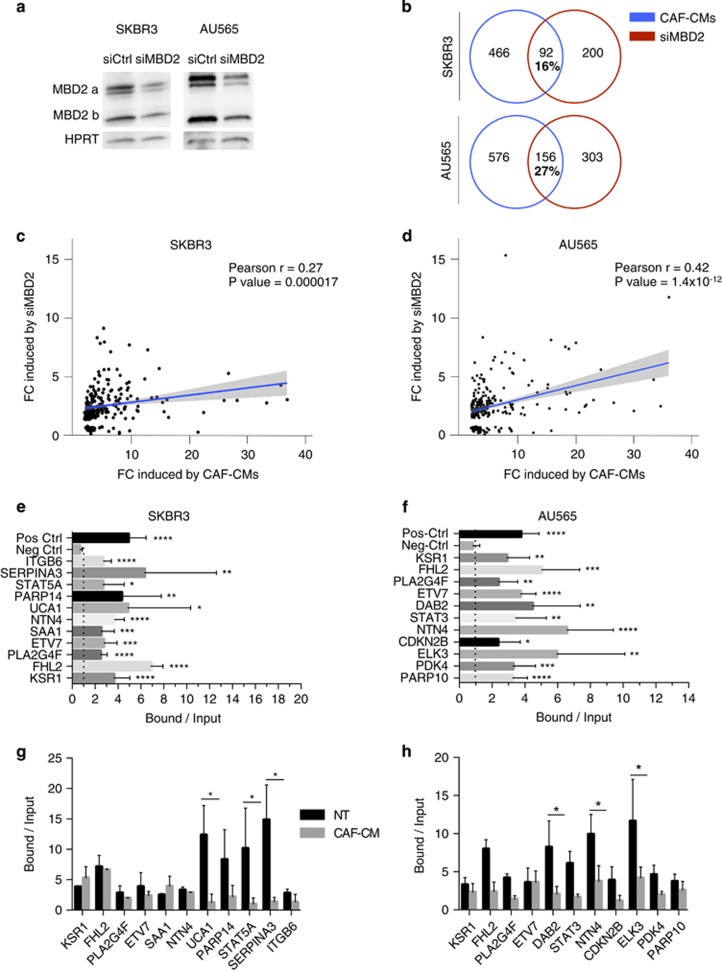
Methyl DNA-binding protein MBD2 deposition at genes modulated by CAF-secreted factors. (**a**) Western blot analysis of MBD2 proteins in SKBR3 and AU565 cells treated with siRNA targeting MBD2. (**b**) Venn diagram of genes upregulated by CAF-CMs compared with genes upregulated by MBD2 siRNA (*P*<0.0001, hypergeometric test). (**c**) Linear regression curve presenting the relationship between the fold change in gene expression induced by CAF-CMs and siRNA targeting MBD2 in SKBR3 and (**d**) in AU565. (**e**) Chromatin immunoprecipitation mapping MBD2-biding sites at the 5’ end regions of genes upregulated by CAF-secreted factors in SKBR3 and (**f**) in AU565. (*P<0.05; **P<0.01; ****P<0.0001, two-tailed unpaired Student’s *t-*test. Error bars=s.e.m). (**g**) Chromatin immunoprecipitation assays of MBD2 enrichment at the 5’ end regions of genes upregulated by CAF-secreted factors in SKBR3 treated with CAF-8-CM (CAF-CM), NT (not treated); and (**h**) in AU565. (*P<0.05; **P<0.01; ****P<0.0001, two-tailed unpaired Student’s *t*-test. Error bars=s.e.m).

Among the proteins involved in the repression of methylated genes, many studies have shown that the methyl-CpG-binding domain proteins (MBD) have a major role in translating DNA methylation into gene repression.^31, 32, 33^ These proteins recognize methylated CpG sites independently of their surrounding sequences and recruit repressor complexes at their binding sites. The MBD2, a member of the MBD protein family, regulates multiple aspects of cell differentiation and function, including immune iPS reprogramming,^34^ immune response^35^ and cancer.^36, 37, 38^ We thus investigated the impact of MBD2 depletion on the set of ‘upregulated genes’. *MBD2* knockdown by transient transfection with a siRNA targeting *MBD2*^39^ led to a 86% and 87% reduction of *MBD2* mRNA in SKBR3 and AU565 cells, respectively, whereas the other members of the MBD family (*MeCP2*, *MBD1* and *MBD4*) were not affected (Supplementary Table S3). This inhibition was also observed at the protein level (Figure 5a).

We identified by RNAseq 292 and 459 genes specifically upregulated in SKBR3 and AU565 cells, respectively, following siMBD2 treatments compared with scrambled control siRNA. A substantial part of these genes were also upregulated by CAF-secreted factors, 16% and 27% in SKBR3 and AU565, respectively (Figure 5b). Furthermore, the fold changes in gene expression observed upon MBD2 depletion were positively correlated with the fold changes induced by CAF-CMs (Figures 5c and d) (Pearson’s *r*=0.27 and *r*=0.42, *P*=0.000017 and *P*=1.4 × 10^−12^ for SKBR3 and AU565 cells, respectively), indicating that a fraction of genes upregulated by CAF-secreted factors are regulated by MBD2 through their DNA methylation pattern. Moreover, MBD2 deposition at 17 genes, upregulated upon MBD2 depletion and treatment with CAF-CMs, was validated by chromatin immunoprecipitation assays (Figures 5e and f) from both cell lines grown in control medium. These data indicated that the methyl-dependent repressor MBD2 targets a part of genes upregulated by CAF-secreted factors. Furthermore, chromatin immunoprecipitation (ChIP) assays indicated MBD2 enrichments were reduced at several upregulated genes, in cell SKBR3 and AU565 cells grown in presence of CAF-secreted factors (Figures 5g and h). Taken together, these data suggest that dynamic MBD2 deposition across methylated DNA regions was associated with the modulation of gene expression by the CAF.

## Discussion

It is well-recognized that some genes are specifically expressed by normal cell of the tumor microenvironment upon the presence of tumor cells.^40, 41, 42, 43, 44^ Concomitantly, the importance of stromal cells for tumor progression has also been established,^17, 43^ although the stroma has been reported to limit cancer progression mainly during the first steps of the oncogenic process.^45^ Dozens of factors secreted by CAFs have now been identified and their role in cancer cell plasticity is widely acknowledged.^21, 46^ The mechanisms underlying the changes in gene expression patterns associated with the cross-talk between cancer cells and the stroma remain an ongoing field of research.

Genetic modifications do not seem to have an important role in the phenotypic modifications observed in fibroblasts upon activation by cancer cells,^47, 48, 49^ however, these phenotypic modifications are conserved over several passages during *in vitro* cultivation of CAFs. Epigenetic modifications should, therefore, have a major role in the cancer–stroma cross-talk.^48^ Epigenetic modifications do not occur only in CAFs but can be induced in cancer cells by CAF interactions because many CAF-secreted factors are involved in epigenetic pathways. For example, the CAF-secreted factor transforming growth factor-β,^22, 50, 51^ mediates epigenetic switches through SOX4 activation, which in turn modulates EZH2, a histone methyltransferase, in cancer cells.^52^ Furthermore, transforming growth factor-β treatments may induce genome-wide changes in DNA methylation, in liver cancer cell lines.^53^ Epigenetic players, such as miRNAs,^54^ also contribute to the maintenance of an invasive, cancer initiating cell phenotype. For instance, loss of Let7 is also expected to induce HMGA2, a regulator of the chromatin remodeling of stemness traits in transformed cells.^55^

Taken together these data indicate that epigenetic markers could have a central role in the tumor–stroma cross-talk. Consistently, inhibition of DNA methylation by exposure to DAC upregulated a large proportion (~60%) of genes upregulated by CAF-secreted factors, identified in a model based on two human breast cancer cell lines.

The analysis of DNA methylation using the Infinium MethylationEPIC beadChip technology, which interrogates 850 000 CpGs, also highlighted the importance of epigenetic marks. The potential regulatory regions (TSS and shore regions) of genes modulated by CAF-secreted factors were hypermethylated when compared with genes unresponsive to these stimuli. We did not detect significant differentially methylated CpG between cells grown in CAF conditioned medium and cells grown in control medium, indicating that gene upregulation driven by CAF-secreted factors was not mediated through changes in DNA methylation. Our results provide evidence that CAF-secreted factors induced phenotypic changes in cancer cell lines without substantial DNA methylation changes. In line with these data, it has been reported that transdifferentiation of B cell to macrophage can occur without DNA methylation changes.^56^ These findings suggest that CAF-secreted factors can induce changes in the interpretation of this epigenetic mark to activate or repress the expression of key genes.

The model used allowed us to isolate the effects of CAF-secreted factors from those occurring during heterotypic interactions, which occur in human tumors. However, we cannot exclude that changes in DNA methylation in cancer cells could be induced by contact with stromal cells in human breast tumors. Alternatively, our model did not allow the identification of the genes undergoing CAF-dependent changes in methylation as cells were grown in CAF culture supernatant for a relatively short time. However, the treatments were long enough to alter the expression of many genes, in the AU565 and SKBR3 breast cancer cell lines, suggesting that long-term DNA methylation changes (including more methylation in certain regions associated with gene upregulation) might more important in locking in transcriptional changes that initiating them. Nevertheless, when clinical breast cancer specimens were classified according to their stromal cell contents (TCGA databank), the methylation patterns of the genes upregulated by CAF-secreted factors did not differ in tumors, exhibiting a high stromal cell content from those with a low stromal cell content.

This epigenetic mark is linked to transcriptional control by various mechanisms. DNA methylation may impair the direct binding of transcription factors to their targets and, in turn, may lead to transcriptional downregulation.^57, 58, 59, 60, 61^ More recently, it has been shown that some transcriptions factors bind to methylated sequences, and for some of them the methylation status of specific CpGs participates in defining their binding sites.^62^ Pioneering studies, from *in vitro* transcription assays, have established that DNA methylation does not inhibit transcription *per se* and suggest that proteins associating DNA sequences play an important role in the repression of methylated templates.^63^ Several families of proteins that recognize methylated DNA with no or weak sequence specificity have been identified.^31, 64^ Their methyl DNA-binding domain has been used to define three major families of proteins,^32, 65^ among them the MBD exhibit weak or no specificities for sequences surrounding methylated CpGs.^65, 66, 67^ Nevertheless, these proteins are involved in many cellular processes. For example MBD2 is a crucial player in differentiation, carcinogenesis, and immunology, and has an important role in the repression of methylated genes, whereas this protein may also act as a transcriptional activator for some genes.^68, 69^ Specific MBD2 deposition occurs at genes downregulated during the *in vitro* transformation of immortalized human mammary cells and for ~10% of them this specific deposition is not driven by changes in DNA methylation but by the redistribution of MBD2 across methylated regions.^39^

In the model used in the present study, MBD2 depletion upregulated ~20% of the genes upregulated by CAF-CMs and chromatin immunoprecipitation (ChIP) experiments have validated the presence of MBD2 at the 5’ end of the 17 genes analyzed. Although these assays were not quantitative enough to monitor potential changes in MBD2 deposition by ChIPseq, ChIP assays indicated that CAF-CMs can reduce MBD2 deposition at several upregulated genes, in SKBR3 and AU565 breast cancer cell lines. A DNA methylation pattern was associated with the genes modulated by CAF-secreted factors, whereas these transcriptomic changes were not associated with direct changes in DNA methylation. These data suggest that MBD2, and other proteins recognizing methylated sequences, may be involved not only in the control of these genes but also in the transcriptomic changes observed upon CAF-secreted factors treatments.

Combining the data obtained from the different cell lines and CAF media used, we identified 372 genes upregulated by CAF-CMs. Many of these genes belong to pathways associated with carcinogenesis and include transcription factors (STAT5A, ETV7, ETV6, SOX4 and SOX9, for example) playing an important role in oncogenesis. In clinical breast tumor specimens, the group of genes identified from cell line models harbored an expression positively correlated with the stromal cell content of the tumors (TCGA databank, *n*=1100), suggesting that these genes were also modulated by the microenvironment in human breast tumors. Furthermore, the stromal cell contents of the tumors did not influence the expression level of genes unaffected (0.8⩽FC⩾1.2) by CAF-secreted factors. Taken together, these data suggested that the *in vitro* model, used in this study, mimicked some characteristics of the cross-talk between tumor cells and stroma, and that the genes identified were involved in the physiopathology of human breast tumors. A DNA methylation pattern, similar to that observed in the AU565 and SKBR3 cell lines, defined the group of stroma-upregulated genes, as they were hypermethylated at their TSS regions compared with the ‘unaffected genes’ or to the ‘other-genes’ groups. Taken together these data suggest that the DNA methylation mark may impact their CAF-dependent expression in human breast tumors. In summary, our study demonstrates that CAF-secreted factors but also stromal content of the tumor remarkably activated specific genes characterized by a DNA methylation pattern. These data provide new insights for the identification of molecular events defining the responsiveness of genes to stromal cell contents of human tumors. These findings might have clinical implications; the targeting of methylation-dependent mechanisms controlling gene expression may represent a new strategy for impacting the effects of stroma on cancer cell plasticity.

## Materials and methods

### Tissue specimens and cultures

Human breast cancer cell lines AU565 and SKBR3 obtained from the American Type Culture Collection were cultured in RPMI-1640 medium (Life Technologies) and McCoy's 5a medium (Life Technologies, Saint-Aubin, France) supplemented with 10% fetal bovine serum (Lonza, Basel, Switzerland) and 1% penicillin/streptomycin (InVitrogen, Carlsbad, CA, USA). After an initial screening using different breast cancer lines (MDA-MB-231, MCF7, T-47D, BT20, MDA-MB-157, SKBR3 and AU565), the AU565 and SKBR3 cell lines were chosen for further studies because they exhibited the most important morphological changes in presence of CAF-secreted factors. Immortalized human mammary epithelial cells were kindly provided by Dr Anne-Pierre Morel (CRCL, Lyon, France) and cultured as previously described.^70^ Primary cultures of breast CAFs were kindly provided by Dr Fréderique SOUAZE (CRCNA, Nantes, France) and cultured in DMEM-F12 (Life Technologies) supplemented with 10% fetal bovine serum and 1% penicillin/streptomycin. Fresh human mammary samples were obtained from chemotherapy naive patients with invasive carcinoma after surgical resection at the Institut de Cancérologie de l’Ouest, René Gauducheau, Nantes, France. All procedures involving patient specimens were approved by the local institutional review boards and all research was carried out in accordance with the Helsinki declaration. CAF samples were obtained with informed consent and human ethics approval from Institut de Cancérologie de l’Ouest (Nantes, France). CAFs were used in the experiments at less than eight passages, and their characteristics are presented in Supplementary Table S1. Cell lines and CAF cultures were tested for mycoplasma monthly using the MycoAlert Mycoplasma Detection Kit (Lonza). To prepare CAF-CM, CAFs were cultured for 48 h in DMEM-F12 supplemented with 1% fetal bovine serum, collected and then centrifuged for 10 min at 1000 rounds per minute to remove cell debris.

### Treatments

For the CAF-CM treatment, cells were seeded at 1.10^5^ per well in six-well plates. The following day, cells were cultured in CAF-CM for 48 h. For MBD2 siRNA experiments, cells were seeded at 3.10^5^ per well in six-well plates. The day after, cells were transfected with 100 pmol of MBD2-targeting siRNA (siMBD2; sense: 5′-GGAGGAAGUGAUCCGAAAdTdT-3′)^39^ or control siRNA (Sigma-Aldrich, MISSION siRNA Universal Negative Controls #1) using RNAiMAX (Sigma-Aldrich, Saint-Quentin-Fallavier, France) as specified by the manufacturer’s instructions. Cells were collected 72 h after treatment initiation. For the DAC treatment, cells were seeded at 1.10^5^ per well in six-well plates. The following day, cells were treated daily with 10 μM of DAC (Sigma-Aldrich) for 3 days and were then collected.

### Immunofluorescence staining

Cells were seeded onto a Nunc Lab-Tek Chamber Slide system (Sigma-Aldrich), and cultured for 48 h prior to their fixation in 3.7% paraformaldehyde for 20 min. Cells were permeabilized in PBS 1 × 0.1% Triton X-100 for 20 min and incubated with the filamentous actin (F-actin) dye (Phalloidin, Fluorescein Isothiocyanate Labeled, Sigma-Aldrich) for 1 h. The cell nuclei were counterstained with 4’,6-diamidino-2-phenylindole, dihydrochloride.

### Expression analysis

Total RNA was extracted using the NucleoSpin RNA kit (Macherey-Nagel, Hoerdt, France) following the manufacturer’s instructions. RNA purity, integrity and quantification were assessed using agarose gel-electrophoresis and analyzed on a NanoDrop 1000 (Thermo Scientific, Courtaboeuf, France). For RNAseq experiments, pools of three independent experiments were sent for high-throughput sequencing to IntegraGen and each experiment was conducted in duplicate. For quantitative real‐time PCR (qPCR), each experiment was carried out in triplicate, 1 μg of RNA was reverse‐transcribed, using the iScript cDNA Synthesis Kit (Bio‐Rad). Quantitative-PCR was performed using a Mini opticon (Bio‐Rad) and the IQ SYBR Green supermix (Bio‐Rad, Marnes-la-Coquette, France). Gene expression profiles were validated using two standard housekeeping genes, *PBGD* and *GAPDH.* Primer sequences are listed in Supplementary Table S4.

### Immunoblotting

Cells were lysed in RIPA Buffer, lysates were sonicated and heated at 95 °C for 5 min. After electrophoresis and transfer, membranes were incubated either with anti-alpha smooth muscle actin (Abcam, ab7817, Paris, France), anti-vimentin (Abcam, ab92547), anti-E-cadherin (BD Biosciences, 610181, Le Pont-de-Claix, France), anti-MBD2a,b (Sigma-Aldrich, RA-18) or anti-HPRT (Abcam, ab10479) antibodies.

### MBD2 ChIP

MBD2 ChIP was performed as previously described.^71^ Sheared chromatin (with a mean fragment length between 300 and 500 bp) was obtained by sonication of formaldehyde crosslinked nuclei. ChIP was then performed with an antibody against MBD2a,b (Sigma-Aldrich, RA-18) using the ChIP Assay Kit (Merck Millipore, Molsheim, France) as specified by the manufacturer’s instructions. Precipitated DNA was purified using the NucleoSpin Gel and PCR Clean-up kit, (Macherey-Nagel) according to the manufacturer’s protocol ‘DNA clean-up of samples containing SDS’. Input and bound fractions of DNA were assayed by fluorometry (Qubit 2.0, Life Technologies). Enrichment in the bound fraction, compared to the input, was measured by qPCR for several genes including one positive (pos-Ctrl) and one negative control (neg-Ctrl), using iQ SYBR Green supermix (BioRad, Marnes-la-Coquette, France). The pos-Ctrl was located at the *BRCA1-NBR2* locus, as this region was methylated in all samples analyzed so far and associated with MBD2 (Auriol *et al.*^71^; Magdinier *et al.*^72^), whereas the neg-Ctrl was a CpG-free region located on chromosome 16 q23.3. Primer sequences are listed in Supplementary Table S4.

### Infinium MethylationEPIC beadChip

Genomic DNA was extracted from cell lines using the QIAmp DNA Mini Kit (Qiagen, Courtaboeuf, France). DNA methylation analysis was subsequently performed for duplicate experiments using the Infinium MethylationEPIC Kit (Illumina, Paris, France) according to the manufacturer’s instructions.^73^

### Statistical and database analyses

For RNAseq analysis, reads were aligned using TopHat2^ref. 74^ on the UCSC *Homo sapiens* hg19 genome. Differential expression was assessed using Cuffdif.^75^

KEGG Analyses were performed using the WEB-based Gene SeT AnaLysis Toolkit (WebGestalt).^24^ For the Infinium MethylationEPIC beadChip analysis, the raw data obtained from the 850 K arrays were processed from the IDAT files with the ChAMP pipeline.^76^ For TCGA analysis, data were downloaded, assembled and processed using TCGA-Assembler^77^ (data download on December 2014). The list of analyzed genes is shown in Supplementary Table S5. Subsequent analyses were performed with the R statistical software v 3.3.1 and GraphPad Prism software.

### Data access

The data set supporting the conclusions of this article is available in the gene expression omnibus repository GSE95462. Private link for the reviewer until acceptance of the manuscript: https://www.ncbi.nlm.nih.gov/geo/query/acc.cgi?token=mvidsgiillsdfux&acc=GSE95462.
