# Supplementary material for: DNA methylation signal has a major role in the response of human breast cancer cells to the microenvironment
Source: Oncogenesis. 2017 Oct 23;6(10):e390–. doi: 10.1038/oncsis.2017.88 (PMC5668886; doi:10.1038/oncsis.2017.88)
Supplement: Supplementary Legends [file oncsis201788x1.docx]

**Supplementary Legends**

**Supplementary Figure S1.** CAFs characterisation. Western blot analysis of α-smooth muscle actin (α-SMA), vimentin (VIM), e-cadherin (E-CAD) and hypoxanthine-guanine ribosyltransferase (HPRT) proteins. A In CAF-8 at different passages (P+5, P+6, P+8) and in Human Mammary Epithelial Cells (HMEC) cell, B In CAF-11 at different passages and in HMEC, C In CAF-12 at different passages and in HMEC, D In CAF-15 at different passages and in HMEC.

**Supplementary Figure S2.** Genes modulated by CAF-secreted factors in SKBR3 and AU565 breast cancer cell lines. Venn diagram of up-and downregulated genes identified by RNAseq in SKBR3 and AU565 cells treated with CAF-CMs

**Supplementary Figure S3.** Characterization of genes upregulated by CAF-CMs in SKBR3 and AU565 breast cancer cell lines. A Venn diagram of upregulated genes identified in the SKBR3 cell line compared to the AU565 cell line. B TOP 10 KEGG pathways contributions by WebGestalt of genes upregulated by CAF-CMs in both AU565 and SKBR3 cell lines (gene number in each pathway > 5).

**Supplementary Figure S4.** Correlation between the expression level of genes upregulated by CAF secreted factors identified *in vitro* and tumors stromal score in human breast infiltrating ductal or lobular carcinoma. A Linear regression curve between the mean expression level of genes upregulated by CAF secreted factors and tumors stromal score in human breast infiltrating ductal carcinoma (n = 752 ). B Linear regression curve between the mean expression level of genes upregulated by CAF secreted factors and tumor stromal score in human breast infiltrating lobular carcinoma (n = 182).

**Supplementary Figure S5.** SKBR3 and AU565 cells exhibited different DNA methylation levels. DNA methylation level (β value) on TSS1500 regions of SKBR3 and AU565 cell lines. (*****P* < 0.0001, two-tailed unpaired Student’s t-test).

**Supplementary Figure S6.** SKBR3 and AU565 cells treated with Decitabine exhibited lower DNA methylation levels. Global DNA methylation level (β value) of A SKBR3 and B AU565 cell lines treated or not with Decitabine (DAC). (*****P* < 0.0001, two-tailed unpaired Student’s t-test).

**Supplementary Figure S7.** Inhibition of DNA methylation have no effect on genes unaffected by CAF-CMs. Venn diagram of genes unaffected by CAF-CMs (0.8 ≤ FC ≥ 1.2) and genes upregulated by DAC treatment (FC ≥ 2) in SKBR3 and AU565 cell lines.

**Supplementary Table S1.** Breast tumors anatomopathological characteristics. Anatomopathological characteristics of the breast tumors where primary cultures of cancer associated fibroblasts (CAF) were established. Oestrogen receptor (ER), Progesterone receptor (PR) and Human Epidermal Growth Factor Receptor-2 (HER2) status were established by Immunohistochemistry.

**Supplementary Table S2.** Correlation between the fold changes induced by the different CAFs . A Person correlation coefficient, r, between the fold changes induced by the different CAF-CMs, in SKBR3. B Person correlation coefficient, r, between the fold changes induced by the different CAF-CMs, in AU565.

**Supplementary Table S3.** Members of the MBD gene family were not affected by the knock down of MBD2 protein. Value in RPKM of *MBD2*, *MECP2*, *MBD1*, *MBD3*, *MBD4* gene, Fold change induced by the siRNA targeting MBD2 protein and q-value from duplicate experiment, analysed with Cuffdif, A in SKBR3 cells and, B in AU565 cells treated with siScramble or siRNA targeting MBD2 protein.

**Supplementary Table S4.** List of primers

**Supplementary Table S5.** List of genes upregulated and unaffected by CAF-CMs in common to SKBR3 and AU565 cell lines.
