# Supplementary material for: DNA methylation signal has a major role in the response of human breast cancer cells to the microenvironment
Source: Oncogenesis. 2017 Oct 23;6(10):e390–. doi: 10.1038/oncsis.2017.88 (PMC5668886; doi:10.1038/oncsis.2017.88)
Supplement: Supplementary Figures [file oncsis201788x2.pdf]

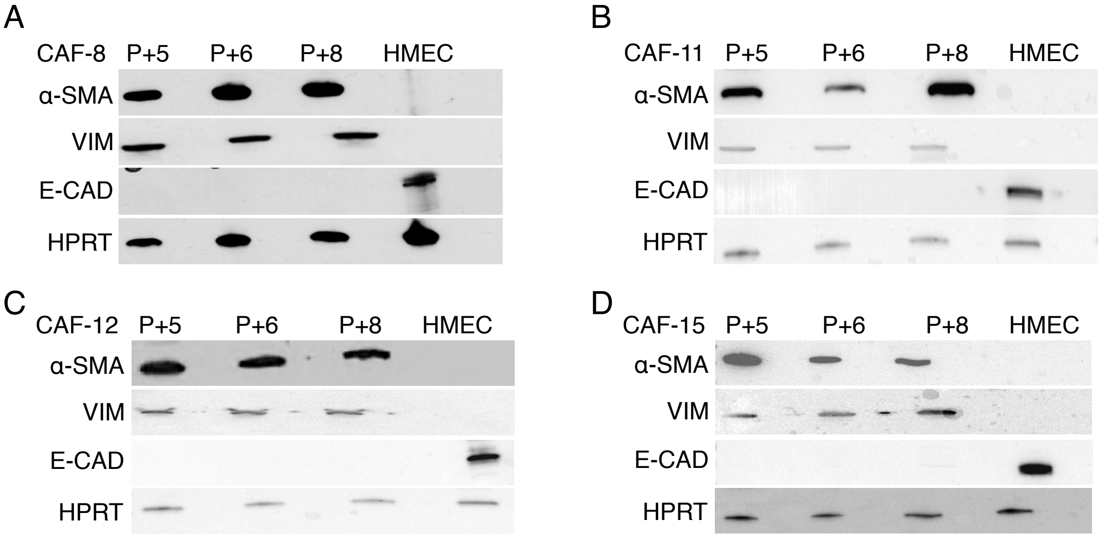

Supplementary Figure S1.

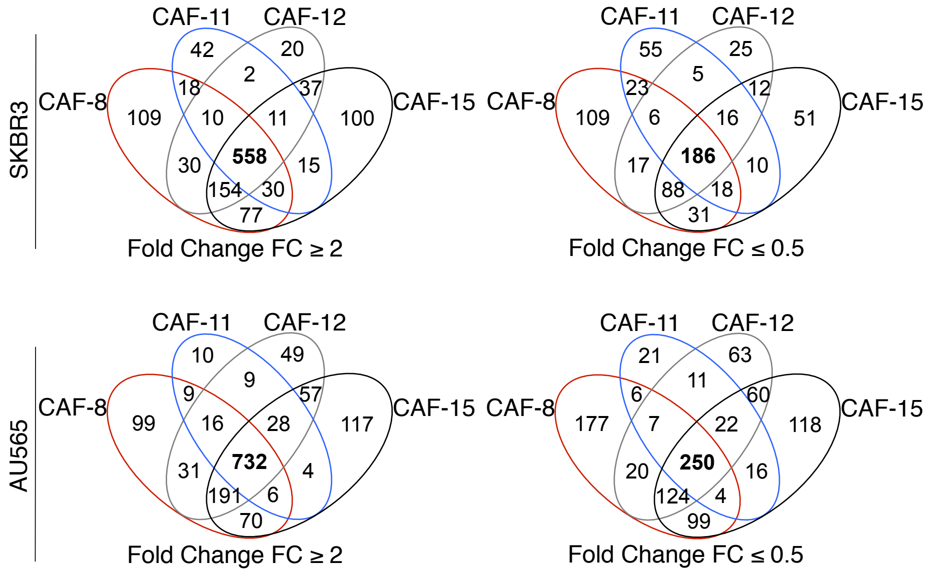

Supplementary Figure S2.

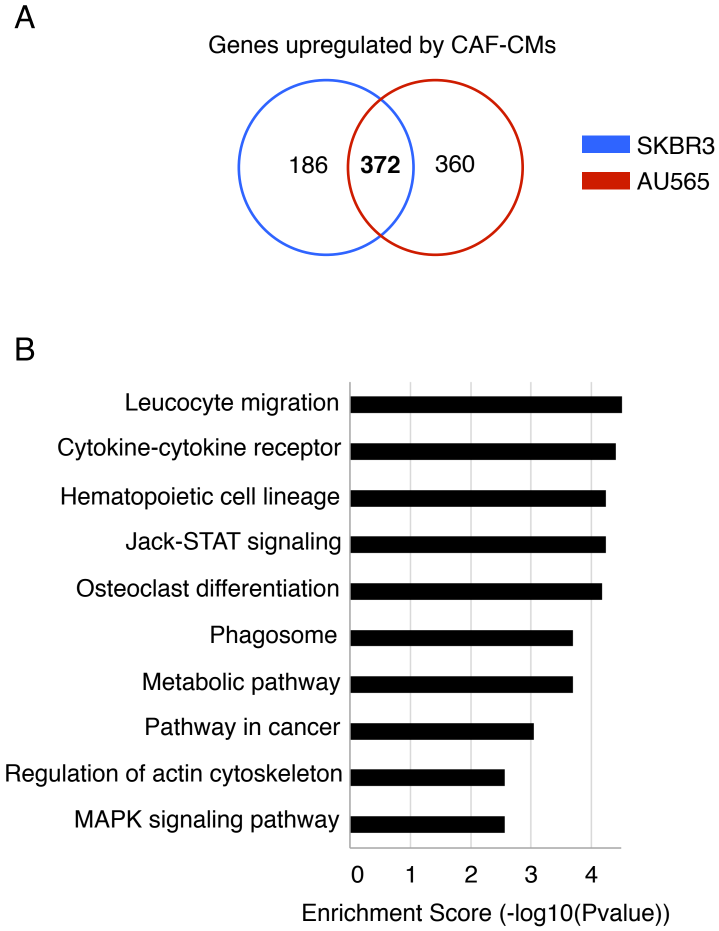

Supplementary Figure S3.

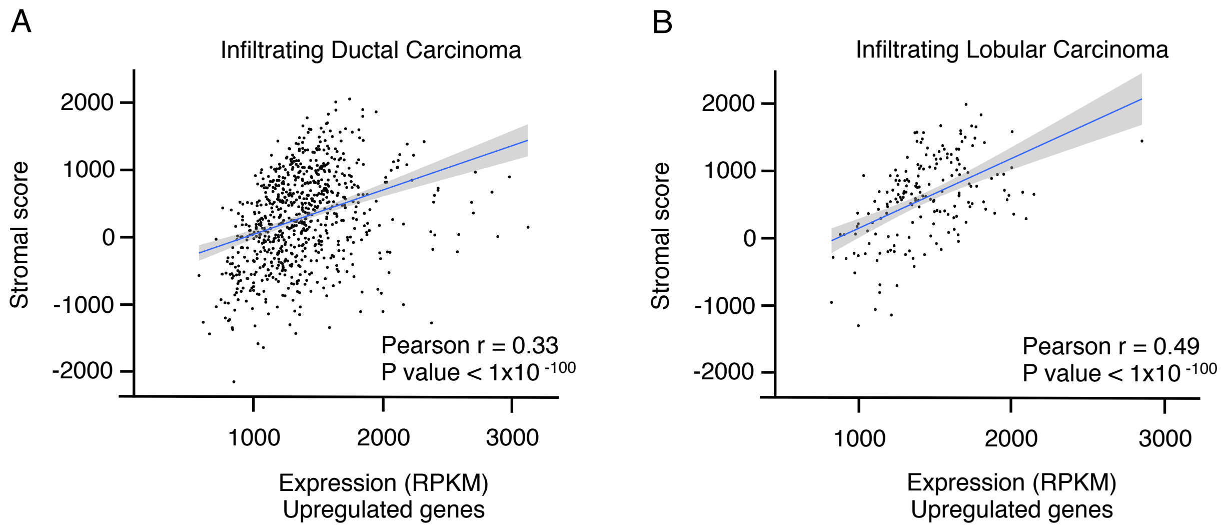

Supplementary Figure S4.

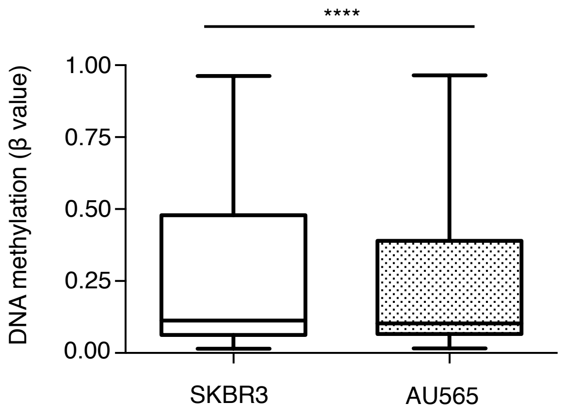

Supplementary Figure S5.

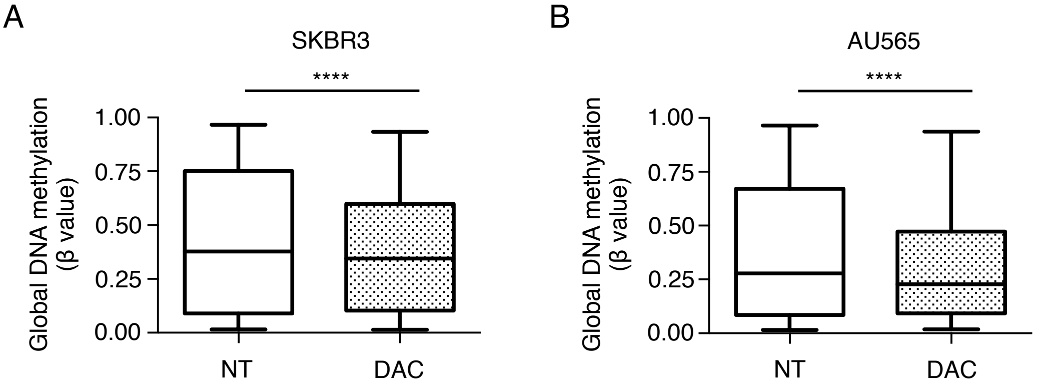

Supplementary Figure S6.

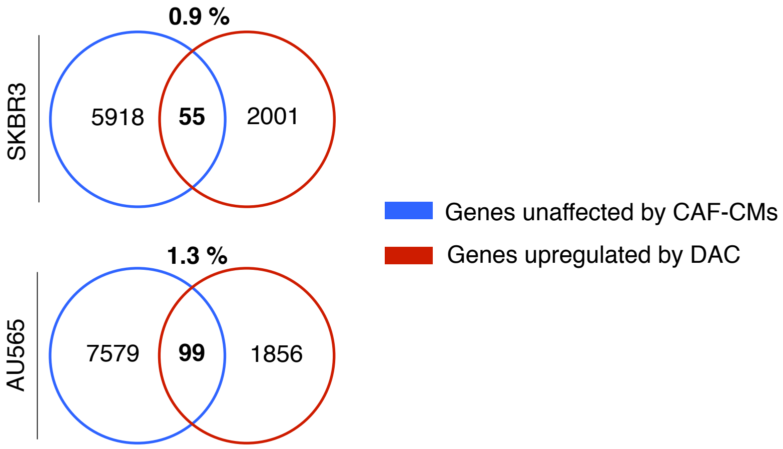

Supplementary Figure S7.
