# Supplementary material for: DNA methylation signal has a major role in the response of human breast cancer cells to the microenvironment
Source: Oncogenesis. 2017 Oct 23;6(10):e390–. doi: 10.1038/oncsis.2017.88 (PMC5668886; doi:10.1038/oncsis.2017.88)
Supplement: Supplementary Tables [file oncsis201788x3.pdf]

CAF	Date	Histological_Type	Grade	Detail_Grade	ER Status	PR Status	HER2 Status
**8**	01/10/13	Infiltrating Ductal Carcinoma	III	3-3-2	ER +	PR +	HER2 -
**11**	19/02/14	Infiltrating Ductal Carcinoma	II	3-3-1	ER +	PR +	HER2 -
**12**	01/11/14	Infiltrating Lobular Carcinoma	II	3-2-1	ER +	PR +	HER2 +
**15**	20/05/14	Infiltrating Ductal Carcinoma	I	2-2-1	ER +	PR +	HER2 -

Supplementary Table S1.

A

SKBR3			
**Pearson r**	CAF-11	CAF-12	CAF-15
CAF-8	0.929	0.978	0.970
CAF-11		0.977	0.960
CAF-12			0.992

B

AU565			
**Pearson r**	CAF-11	CAF-12	CAF-15
CAF-8	0.942	1	0.990
CAF-11		0.942	0.902
CAF-12			0.989

Supplementary Table S2.

A

SKBR3

Gene	Value in cells treated with siScramble	Value in cells treated with siMBD2	Fold change	q-value	Significant
MBD2	12.2	1.6	0.1	0.0021	yes
MECP2	11.9	10.3	0.9	0.7721	no
MBD1	14.4	13.8	1.0	0.9361	no
MBD3	21.9	22.1	1.0	0.9895	no
MBD4	14.7	13.6	0.9	0.8812	no

B

AU565

Gene	Value in cells treated with siScramble	Value in cells treated with siMBD2	Fold change	q-value	Significant
MBD2	14.0	1.8	0.1	0.0013	Yes
MECP2	13.4	11.8	0.9	0.7832	no
MBD1	14.8	14.8	1.0	0.9990	no
MBD3	9.4	11.8	1.3	0.1893	no
MBD4	15.3	14.7	1.0	0.9453	no

Supplementary Table S3.

qPCR Primers	Forward primer (5' to 3')	Reverse primer (5' to 3')
ITGB6	ATC-GGT-CTG-CAC-AGC-AAG-AA	CAG-GCA-CAC-TGA-GGT-CCA-AT
FHL2	CTC-ATC-CAA-GTG-CCA-GGA-AT	GTG-GCA-GAT-GAA-GCA-GGT-CT
STAT5A	CCC-CCA-GGC-TCC-CTA-TAA-CA	AGA-GGT-GAA-AAG-ACC-GGC-AG
MUC20	AAG-ACC-TCA-CTG-ACC-CCA-GA	ACC-TCT-CAG-CAC-AGT-AAC-GC
SERPINA3	CTT-CTC-CAG-CTG-GGC-ATT-GA	GGC-CTG-TTG-AAA-CGC-ACA-AT
SAA1	ATG-ATG-CTG-CCA-AAA-GGG-GA	CCA-CTC-CTG-CCC-CAT-TCA-TT
MBD2	TCA-GAA-GCA-AGC-CTC-AGT-TG	CAG-AGC-TTG-TGT-GCA-AAG-CA
GAPDH	CGG-AGT-CAA-CGG-ATT-TGG-TCG-TAT	AGC-CTT-CTC-CAT-GGT-GGT-GAA-GAC
PBGD	GAG-TGA-TTC-GCG-TGG-GTA-CC	GGC-TCC-GAT-GGT-GAA-GCC
**ChIP qPCR Primers**		
ITGβ6	GAG-TTA-GCA-AGC-TCC-TGG-CA	CTC-CCG-GCT-TTC-CAA-AGA-GA
SERPINA3	GGA-GGA-GGG-ACA-ACC-ACT-TG	CAC-CTA-GAG-AGG-GGT-GTG-GA
STAT5A	CTG-AAG-GGA-ACT-GCT-GGA-GG	AAT-GGA-TTT-GGC-AGG-GGA-GG
PARP14	GAG-CAC-CTA-TTG-CCC-ACT-GT	TCA-CAA-ACT-GTG-GCC-CCT-TT
UCA1	TCA-CTG-TGT-GAG-GGT-TTC-GG	TCA-GGA-CTT-GGC-AAC-ACC-AA
NTN4	CTC-GGC-TAA-ACA-ATC-GCT-GC	TTA-GCT-GCG-TGT-GGT-TGC-TA
SAA1	GGT-GGT-AAC-TCC-TGC-CTT-CC	CCC-GTG-AGA-AGC-TTC-ATG-GT
ETV7	ACC-TAG-AAA-ACC-GGC-GAG-TG	CAC-TCA-CTT-CCC-CTT-CCA-CC
PLA2G4F	GCC-TTA-GGC-TGC-AGA-TGG-AT	TAG-GCC-TTG-GGA-AGA-GTG-GA
FHL2	GGA-AGG-TCC-TAT-CCC-CAC-CT	CTC-AAC-CTG-TGC-TCT-CCC-TG
KSR1	GCA-CCA-ACT-CAG-CAA-ACA-GG	TTA-CCA-CGG-TGA-AGG-CTG-AC
DAB2	TCG-GGG-AGA-AGT-CAA-AAG-CC	AGG-AAC-CGT-TGT-CCC-TTG-TC
STAT3	TCT-TAC-CAC-GCG-GGA-ATC-AG	ATG-TTT-CCG-GGT-GTG-TGT-GT
CDKN2B	TCG-CGA-AGC-AAG-TTG-ACT-GA	GGG-AGA-AAA-GGG-GCT-TTG-GA
ELK3	ACC-GAA-AAC-CCA-GAC-AGG-AA	GCT-GGG-ATG-GAA-GAG-TTG-GG
PDK4	GGG-GCT-GCC-CAG-ATA-CAT-TT	GAC-CTT-TTG-CAG-CAG-ACA-GC
PARP10	CCC-CTC-ACT-CAG-GAT-CTC-CA	CAG-GAC-AGA-AAG-CTG-CAG-GA
Pos-Ctrl	AAG-GGC-TCC-TCC-AGC-ACG-GC	TTC-TGA-GGG-ACC-GAG-TGG-GC
Neg-Ctrl	GAA-GGC-TGC-ATG-GAA-ATG-AT	GGA-TCT-GGA-TCG-GAA-GGA-AC

Supplementary Table S4.
