# Supplementary material for: DNA methylation signal has a major role in the response of human breast cancer cells to the microenvironment
Source: Oncogenesis. 2017 Oct 23;6(10):e390–. doi: 10.1038/oncsis.2017.88 (PMC5668886; doi:10.1038/oncsis.2017.88)
Supplement: Supplementary Information [file oncsis201788x4.pdf]

Liste of Upregulated genes in common to SKBR3 and AU565
LBP
S100A7
TGM2
CP
S100A7A
SERPINA3
LCN2
MUC4
STEAP4
NNMT
CSF3R
ICAM1
PIGR
PDZK1IP1
SLC28A3
FAM83A
S100A8
CAMP
SOCS3
SAA1
CES1
RARRES3
PTGS1
C1S
CNDP1
LYPD5
SAA2
FGA
C2CD4A
C10orf10
PHLDB2
MUC20
PRDM1
IL22RA2
IFITM1
AKR1B10
GPR97
C1R
TMC5
A4GALT
B3GNT7
CASP10
LRG1
SORBS1
CYP4F11

PARP9
MAN1A1
PLEKHS1
APOL1
ELOVL3
CTSS
HPX
CASP1
PROM1
CHI3L1
BHLHE40
NFE2
LGALS9
UCA1
IL1R1
CD36
PLIN5
GDPD1
AKR1B15
IFITM3
GBP2
ABCA1
CD14
FMO3
APOL6
SLC7A2
UBE2L6
HCAR2
CLCA2
TNFRSF14
KLHDC7B
PARP14
MMP13
TRIM22
NLRC5
PTPRE
COL27A1
BST2
RASL11A
C19orf66
C1RL
HPGD
MRVI1
TEAD2
FGL1
A2M

SUSD3
GSDMC
NTN4
ITGB2-AS1
NFAM1
DTX3L
TRANK1
CASP14
CLEC7A
FAM78A
CHRD
TMEM173
CEBPD
UBA7
ACOX2
GBP3
STAT5A
LOC100133669
KCNK3
LOC284751
HAPLN3
ARL4D
LINC00673
PDGFRL
PRKAR2B
FUT2
TMPRSS4
WNT4
GPR110
VLDLR
TFPI2
HES2
GUCY1B3
GLYATL2
PDK4
SLC2A10
SLFN5
CSF1
C1QTNF1
TLR5
ERAP2
DHRS3
HSD17B14
PCSK6
GGT5
BATF2

DDX60
DTX4
SLC16A5
GBP1
CYP4Z1
FUT3
CARD6
NFKBIZ
VWA2
IRF1
FAM131B
SOX9
CASP4
GLRX
HERC6
FHL2
SGK1
EPHB2
ARAP3
TRIB1
DUSP27
DNAH17
SLC12A4
FTH1
BPIFB1
TAPBPL
ITGB6
KSR1
MXRA5
PPAP2C
RAC2
EYA2
ITGB2
STAC2
DAPK2
C1orf228
MCF2L
IL1R2
GPR37L1
SLC1A6
MSMB
ADM
IRF9
TCHHL1
ST6GAL1
PPP1R1B

NRG2
RORC
SERPINB1
NCOA7
GPRC5B
SLC43A2
SLC35C1
RIN3
INSR
CATSPERB
ST5
BCL6
FAM198A
SNAI1
PCDH20
SERPINA1
ACTBL2
TTC39B
ATP2B4
SLC6A6
PARP10
YPEL2
SEMA4B
NAALADL2
ZSWIM4
CCL2
CADPS2
UBASH3B
DHX58
LOC100129550
MDK
PSMB10
DLL4
VWA1
TMEM2
APOD
ABCC4
BTN3A1
RAPGEF3
ABCA4
HOMER3
DDR2
CSTA
ZNF117
ORAI2
DPYD

IFI35
DAB2
PGM2L1
TSKU
BMF
CEACAM6
SIPA1
TLE6
DDIT4
RCN1
BIN1
WDR91
LOXL2
GPRC5A
NINL
PANX2
SHROOM1
FGD3
PLEKHG7
MSRB1
INPP4B
TCHH
SH3TC1
SOX4
DUSP10
IL2RB
GYG2
PFKFB3
FOLR1
TUBB3
SUSD4
TLE2
C14orf182
MAP3K8
RHBDL2
PLA2G4F
TLCD2
ARHGDIB
CEBPB
TP53INP1
CD68
NPAS2
ELK3
PCDH1
MUC1
EPDR1

MZB1
DNAH5
LOC100506810
AFAP1L2
ERV3-1
MYLK
UGCG
PTGES
VWA5A
CYP4X1
CDRT4
ETV7
CTGF
SCCPDH
SNX33
ETV6
WISP1
ITPR1
SAMD9
HDAC7
KITLG
LRRC8C
TNFAIP8
RPGRIP1
MICAL1
ATHL1
ARRDC3
RNF24
MTHFR
TRIM38
ERP27
MVP
ALPK1
JUNB
SYTL2
AARD
TMEM45A
TPST1
B4GALT5
SAMD12
GADD45B
IFITM2
DDIT4L
IL15
PTPRH
CCDC146

PLCL2
PLEKHG2
TRIM6
PLD1
MAPK13
ZCCHC24
PRSS22
TSPAN15
AUTS2
BCL3
CDH5
RUNX1
FAIM3
C9orf84
EFNA5
CCR7
ZNF703
XYLT1
IL13RA1
SLCO2A1
GALNT16
ELF3
CAPN13
MAST4
SDCBP
MYO18A
NCF4
CLINT1
GIPR
RIT1
C1orf226
RHOBTB1
ACPP
NRP1
STAT3
CNP
SIX5
TH
HIF1A
FNDC3B
FLJ45445
PLSCR1
GFOD2
IRF7
ITGAL
SLC16A4

ADORA1
TSPAN3
TCP11L2
DLGAP1-AS1
SLC34A3

Liste of Unaffected genes in common to SKBR3 and AU565
AAGAB
AAMP
AAR2
AARS
AARS2
AASDH
ABCD3
ABCF1
ABCF3
ABHD11
ABHD13
ABHD16A
ABHD3
ABHD5
ACAD10
ACAD11
ACADVL
ACAP2
ACIN1
ACOT13
ACOT9
ACP6
ACSF3
ACTR10
ACTR1A
ACTR1B
ACTR2
ACTR3
ACTR3B
ACTR6
ACVR1B
ACVR2A
ADAM3A
ADAM5
ADAMDEC1
ADAT1
ADCK1

ADCYAP1
ADD1
ADH1A
ADH5
ADNP
ADNP2
ADO
ADSS
AFG3L1P
AGAP11
AGER
AGGF1
AGL
AGO1
AGPAT6
AHCYL1
AHCYL2
AHI1
AHNAK
AIF1
AK3
AKAP10
AKAP11
AKAP8L
AKIRIN2
AKT2
ALAD
ALAS1
ALDH18A1
ALDH2
ALDH9A1
ALG1
ALG13
ALG1L2
ALG3
ALG5
ALG9
ALKBH4
ALKBH5
AMMECR1
AMY1B
AMZ2P1
ANAPC16
ANAPC2
ANGEL1
ANGEL2

ANKEF1
ANKFY1
ANKIB1
ANKLE2
ANKRD13C
ANKRD17
ANKRD20A1
ANKRD20A2
ANKRD20A3
ANKRD26
ANKRD40
ANKRD52
ANP32A
ANXA2P2
ANXA5
ANXA7
AP1AR
AP2M1
AP3M1
AP4B1
AP4E1
AP4M1
AP5M1
APBB2
APC
APEX2
APH1A
API5
APLP2
APOC4
APOM
APOOL
APPL1
AQR
ARAF
ARFGAP2
ARFIP1
ARFIP2
ARFRP1
ARHGAP1
ARHGAP8
ARHGEF2
ARHGEF7
ARHGEF9
ARID1B
ARID2

ARIH1
ARIH2
ARL16
ARL3
ARL5A
ARL6IP4
ARL6IP5
ARMC8
ARPC1A
ARPC2
ARPC4
ARPC5
ARPC5L
ARPP19
ASB1
ASB18
ASB8
ASCC2
ASIC3
ASNSD1
ASTE1
ASXL1
ATAD2B
ATAT1
ATE1
ATF1
ATF2
ATF5
ATF6B
ATG12
ATG2B
ATG4B
ATIC
ATP11B
ATP13A3
ATP1A1
ATP1B4
ATP2B1
ATP5SL
ATP6AP2
ATP6V1A
ATP6V1B2
ATP6V1D
ATP6V1G2
ATPAF2
ATXN10

ATXN1L
ATXN2
ATXN7
AUH
AUP1
AVEN
AVP
AZU1
B3GALT4
B4GALT3
BABAM1
BAG3
BAG5
BAG6
BAK1
BANF2
BAP1
BBS1
BBS10
BBS4
BBS9
BCAP29
BCAP31
BCAR3
BCAS2
BCKDK
BCL2L11
BCL2L13
BCL2L2
BCL7B
BCYRN1
BECN1
BIRC2
BLCAP
BLMH
BLOC1S5
BLOC1S6
BLVRB
BLZF1
BMPR1A
BMS1
BMX
BNIP2
BPGM
BRAP
BRCC3

BRD7
BRD9
BRF2
BRK1
BRPF1
BRWD1
BSG
BSPH1
BSX
BTAF1
BTBD9
BTNL2
C10orf32
C10orf35
C10orf53
C10orf76
C10orf88
C11orf30
C11orf40
C11orf49
C11orf57
C11orf58
C12orf29
C12orf39
C15orf40
C15orf43
C16orf92
C17orf105
C17orf50
C17orf80
C17orf85
C18orf21
C18orf25
C18orf8
C19orf52
C1orf112
C1orf146
C1orf172
C1orf27
C1orf43
C1orf50
C1orf52
C1orf74
C2
C21orf54
C22orf29

C22orf39
C2orf27B
C2orf42
C2orf47
C2orf68
C2orf80
C3orf17
C3orf38
C3orf62
C3orf79
C4A
C4B
C4orf29
C4orf51
C5orf22
C5orf24
C5orf51
C5orf52
C5orf55
C6orf10
C6orf106
C6orf120
C6orf132
C6orf136
C6orf15
C6orf211
C6orf25
C6orf47
C6orf48
C6orf89
C7orf13
C7orf26
C7orf66
C8orf33
C9orf156
C9orf69
CAAP1
CAB39
CABLES2
CABP5
CACTIN
CAMKK2
CAMLG
CAMSAP1
CAMSAP2
CAMTA1

CAPN10
CAPN5
CAPN7
CAPRIN1
CAPRIN2
CAPZA1
CAPZA2
CAPZB
CARM1
CARS
CASK
CBFB
CBLL1
CBX1
CBX7
CCAR1
CCDC117
CCDC12
CCDC132
CCDC137
CCDC167
CCDC174
CCDC23
CCDC25
CCDC47
CCDC6
CCL1
CCL11
CCL16
CCL18
CCL19
CCL21
CCL24
CCL3L3
CCL4L1
CCL7
CCND3
CCNE1
CCNG1
CCNI
CCNK
CCNT1
CCNT2
CCNYL1
CD151
CD164

CD2AP
CD2BP2
CD58
CD82
CDAN1
CDC16
CDC23
CDC27
CDC37
CDC37L1
CDC40
CDC5L
CDC73
CDK12
CDK16
CDK17
CDK7
CDK8
CDKN1B
CDSN
CDYL
CEBPG
CELF1
CEP135
CEP192
CEP41
CEP44
CEP57
CEP63
CEP85
CEPT1
CERCAM
CERS5
CFC1B
CFL1
CGB1
CGB2
CGB5
CGGBP1
CGRRF1
CHCHD2
CHD1
CHD4
CHERP
CHMP2B
CHMP3

CHMP4B
CHMP7
CHODL-AS1
CHRAC1
CHSY1
CHTF8
CHTOP
CHUK
CHURC1
CIRBP
CISD2
CLASP2
CLASRP
CLC
CLCC1
CLCN3
CLDN15
CLDN22
CLDN7
CLDND1
CLEC2A
CLEC4E
CLIC1
CLIP1
CLK4
CLN8
CLP1
CLPP
CLPTM1
CLPTM1L
CLPX
CLSTN1
CLTA
CLTC
CMAS
CMPK1
CNBP
CNEP1R1
CNNM3
CNOT1
CNOT11
CNOT2
CNOT4
CNOT6
CNOT7
CNPY2

CNPY3
COA3
COA4
COA5
COG1
COG2
COG5
COG7
COG8
COIL
COL11A2
COL4A3BP
COPG2
COPS2
COPS5
COPS6
COPS7B
COPZ1
COQ4
COX14
COX15
COX19
COX8C
CPEB3
CPLX3
CPNE1
CPNE3
CPSF2
CPSF3
CPSF4
CPSF6
CPSF7
CRCP
CREB1
CREBL2
CREBZF
CREM
CRK
CRLF3
CROCCP2
CRTAP
CRYGB
CRYZL1
CSAG1
CSDE1
CSGALNACT2

CSH1
CSN1S2BP
CSNK1A1
CSNK1D
CSNK2A1
CSNK2A2
CSNK2A3
CSNK2B
CST13P
CSTF1
CT45A1
CT45A6
CT47A1
CT47A6
CTBP1
CTCF
CTDNEP1
CTNNA1
CTR9
CTSL3P
CUL1
CUL2
CUL3
CUL4A
CUL4B
CUL5
CUTA
CWC22
CWC25
CXorf23
CXorf38
CXorf40A
CXorf40B
CXorf56
CXXC1
CYB561
CYHR1
CYMP
CYP20A1
CYP21A2
CYP2U1
DAAM1
DAD1
DAG1
DAGLB
DAP3

DAPK3
DARS
DAZAP1
DBT
DCAF10
DCAF11
DCAF15
DCAF4
DCAF7
DCAF8
DCD
DCP2
DCTD
DCTN2
DCTN4
DCTN5
DCTN6
DCUN1D1
DDA1
DDAH2
DDB1
DDHD1
DDR1
DDRGK1
DDX19B
DDX23
DDX24
DDX28
DDX39B
DDX42
DDX47
DDX5
DDX59
DDX6
DECR1
DEFA10P
DEFA1B
DEFA3
DEFA4
DEFA6
DEFB104B
DEFB105A
DEFB108B
DEFB113
DEFB115
DEFB116

DEFB122
DEFB123
DEFB124
DEFB131
DEFB133
DEFB134
DEFB135
DEFB136
DENND1B
DENND1C
DENND5A
DENND6A
DERA
DET1
DEXI
DGAT1
DGAT2L6
DGCR8
DGUOK
DHPS
DHX16
DHX29
DHX37
DHX38
DHX40
DIABLO
DIAPH1
DICER1
DIP2A
DIP2B
DIS3
DKFZp434L192
DKFZp566F0947
DKFZP586I1420
DLG1
DMAP1
DNAI2
DNAJA1
DNAJA2
DNAJB12
DNAJB4
DNAJC13
DNAJC15
DNAJC25
DNAJC27
DNAJC4

DNAJC5G
DNAJC8
DNAL4
DNASE1
DND1
DNPEP
DOCK3
DOCK9
DPAGT1
DPCR1
DPF2
DPM2
DPM3
DPY19L3
DPY19L4
DPY30
DRG2
DROSHA
DSCR3
DSP
DST
DSTYK
DTNB
DUSP11
DUSP12
DUSP3
DVL3
DYNC1LI1
DYNLL2
DYNLRB1
DYNLT1
DYRK1A
E2F4
E2F6
EAF1
EAPP
EBPL
ECI1
EDC3
EDF1
EED
EFCAB1
EFCAB2
EFCAB7
EFHD2
EFR3B

EFTUD2
EGFL8
EHBP1
EHF
EHMT1
EHMT2
EIF1AD
EIF1B
EIF2A
EIF2AK1
EIF2AK4
EIF2B5
EIF3A
EIF3G
EIF3I
EIF4A1
EIF4A2
EIF4E2
EIF4EBP2
EIF4ENIF1
EIF4G1
EIF4G2
EIF4H
EIF5
EIF5B
ELAC1
ELAVL1
ELF2
ELK1
ELMOD2
ELP3
ELP4
ELP5
ELSPBP1
EMC1
EMC3
EMC4
EMX2OS
ENTPD6
EPB41L5
EPCAM
EPHB3
EPM2AIP1
EPN2
EPS15
EPS8L1

EPS8L2
ERCC1
ERCC3
ERCC6
ERGIC3
ESCO1
ESRP1
ESYT2
ETAA1
ETF1
ETNK1
EWSR1
EXOC1
EXOC2
EXOC5
EXOC8
EXOSC1
EXT2
EXTL3
EYA3
F11R
FABP9
FADD
FAHD1
FAM104A
FAM114A2
FAM115A
FAM117B
FAM118A
FAM120A
FAM120B
FAM124A
FAM134A
FAM135A
FAM138B
FAM13B
FAM149B1
FAM153B
FAM159A
FAM160A2
FAM163B
FAM173B
FAM175B
FAM178A
FAM189B
FAM193A

FAM195B
FAM197Y2
FAM199X
FAM19A1
FAM206A
FAM208A
FAM217B
FAM219B
FAM32A
FAM49B
FAM50A
FAM53C
FAM73A
FAM76B
FAM83H
FAM8A1
FAM96A
FAM98A
FAM99A
FASTK
FASTKD3
FASTKD5
FBRSL1
FBXL17
FBXO11
FBXO18
FBXO21
FBXO22
FBXO3
FBXO30
FBXO31
FBXO33
FBXO38
FBXO42
FBXO7
FBXW4
FBXW7
FEM1C
FERMT3
FGF16
FHDC1
FIP1L1
FKBP14
FKBPL
FLII
FLJ10038

FLJ31306
FLJ46361
FLOT1
FMR1
FNBP1L
FNBP4
FNTA
FOXJ2
FOXK2
FOXN3-AS2
FPGS
FRAT2
FRG1B
FRG2
FRG2C
FRS2
FRYL
FTO
FUBP3
FUCA2
FUK
FUNDC1
FUNDC2
FUT1
FUZ
FXR1
FYCO1
FYTTD1
FZD3
G3BP1
G3BP2
GABBR1
GABPA
GAGE10
GAGE12G
GAGE2A
GAGE2C
GAGE2D
GAGE2E
GAGE5
GALE
GALK2
GALNT3
GALT
GAN
GAPDH

GAPVD1
GAS2L1
GATAD2A
GATAD2B
GATC
GBE1
GCA
GCDH
GCH1
GCLC
GDI1
GEMIN7
GFER
GFM2
GGA1
GGA2
GGH
GGNBP2
GGPS1
GHDC
GHITM
GHRH
GID8
GIGYF2
GIN1
GINM1
GIP
GIT2
GK5
GLA
GLTSCR1L
GLUL
GLYR1
GMPR2
GNA12
GNA13
GNAI3
GNB1
GNB2L1
GNG5
GNG8
GNL1
GNL3
GOLGA4
GOLGA7
GOPC

GORASP1
GOSR1
GPANK1
GPATCH1
GPATCH3
GPATCH8
GPBP1
GPBP1L1
GPD1L
GPI
GPKOW
GPR180
GPSM3
GPT2
GPX5
GRB2
GRP
GRSF1
GSC2
GSE1
GSK3A
GSPT1
GSS
GSTO1
GSTTP2
GTF2E2
GTF2H1
GTF2H3
GTF2H4
GTF3C2
GTF3C4
GUCA2B
GXYLT1
GYG1
GYPB
GYS1
GZF1
GZMK
H3F3B
HACE1
HAGH
HAR1B
HARS
HARS2
HAUS6
HCG18

HCG22
HCG26
HCG27
HCG4
HCG9
HCP5
HDAC1
HDAC3
HDAC8
HDHD2
HEATR3
HEATR5B
HEATR6
HEBP2
HECTD1
HELQ
HERC2
HES3
HEXB
HHATL
HIATL1
HIF1AN
HIGD1A
HIGD1C
HIGD2A
HIPK1
HIRA
HIST2H2AA3
HKR1
HLA-DMB
HLA-DOA
HLA-DOB
HLA-DPA1
HLA-DPB1
HLA-DPB2
HLA-DQA2
HLA-DQB2
HLA-DRA
HLA-DRB6
HLA-F
HLA-G
HLA-J
HLA-L
HLCS
HMGXB4
HNRNPA0

HNRNPF
HNRNPK
HNRNPUL1
HOOK3
HOXD11
HP1BP3
HPS4
HPS6
HPVC1
HPYR1
HS2ST1
HSBP1
HSD17B12
HSD17B8
HSF1
HSFX2
HSPA1A
HSPA1L
HTATSF1
HTN3
HTRA2
HTT
HUS1
HYALP1
IDE
IDH3G
IER3IP1
IFFO2
IFI30
IFIH1
IFITM4P
IFT20
IFT52
IFT80
IFT81
IFT88
IK
IKZF5
IL22
IL26
IL36B
IL9
ILF2
ILF3
IMPAD1
ING1

ING3
INPP4A
INPP5E
INPP5K
INTS10
INTS4
INTS5
INTS7
INTS8
INVS
IP6K1
IQCB1
IQCG
IREB2
ISCA2
ISOC2
IST1
ITCH
ITFG2
ITFG3
ITGAV
ITPA
JKAMP
JMJD7
JMJD8
JOSD1
KANSL1L
KANSL2
KANSL3
KAT2A
KAT5
KAT7
KAT8
KATNB1
KATNBL1
KBTBD2
KBTBD7
KCMF1
KCNJ14
KCNN4
KCNQ1DN
KCTD18
KCTD2
KDM1A
KDM2A
KEAP1

KHDRBS1
KHDRBS2
KHSRP
KIAA0040
KIAA0100
KIAA0141
KIAA0196
KIAA0368
KIAA0895L
KIAA1033
KIAA1147
KIAA1191
KIAA1279
KIAA1324L
KIAA1467
KIAA1598
KIAA1715
KIAA1841
KIAA2013
KIDINS220
KIF16B
KIF3B
KIF5B
KIFAP3
KIFC1
KIFC2
KIN
KIR2DL4
KIR3DL2
KLC4
KLF12
KLF16
KLF3
KLF7
KLF9
KLHDC3
KLHL28
KLHL8
KLHL9
KLK4
KLKP1
KPNA1
KPNA6
KRAS
KRBOX4
KRI1

KRIT1
KRR1
KRTAP10-6
KRTAP12-4
KRTAP19-3
KRTAP19-4
KRTAP19-8
KRTAP2-1
KRTAP2-2
KRTAP2-4
KRTAP20-1
KRTAP20-4
KRTAP21-1
KRTAP21-3
KRTAP22-1
KRTAP22-2
KRTAP23-1
KRTAP4-5
KRTAP5-3
KRTAP5-4
KRTAP5-5
KRTAP6-2
KRTCAP2
KRTDAP
KTI12
KXD1
L3MBTL2
LACRT
LACTB
LALBA
LAMP1
LAMTOR2
LAMTOR3
LAMTOR4
LAMTOR5
LANCL2
LARP1
LARP1B
LARP4
LARP4B
LARP7
LATS2
LCE1A
LCE1D
LCE1E
LCE2B

LCE2C
LCE2D
LCE4A
LCE6A
LCMT1
LCN1
LCN6
LCN9
LDB1
LDHA
LDOC1L
LEPROTL1
LETMD1
LGALS7
LGALS7B
LGMN
LHPP
LIMD1
LIMS1
LIN54
LIN7C
LINC00113
LINC00114
LINC00159
LINC00161
LINC00207
LINC00238
LINC00261
LINC00301
LINC00320
LINC00339
LINC00474
LINC00488
LINC00575
LINC00588
LMBR1
LOC100129935
LOC100133050
LOC100169752
LOC100190940
LOC100192426
LOC100287834
LOC144742
LOC145474
LOC146481
LOC148145

LOC148413
LOC154449
LOC220729
LOC255025
LOC283856
LOC283914
LOC284276
LOC284688
LOC284788
LOC285627
LOC285696
LOC286135
LOC339568
LOC339788
LOC340074
LOC340094
LOC340357
LOC389033
LOC389332
LOC400657
LOC493754
LOC606724
LOC643923
LOC644669
LOC646498
LOC648691
LOC727677
LOC729121
LOC730811
LOC731779
LOC732275
LPPR2
LRRC28
LRRC37BP1
LRRC42
LRRC47
LRRC57
LRRC59
LRRC8E
LRRFIP2
LRSAM1
LSM14A
LSM14B
LSM2
LST1
LTA

LTA4H
LTB
LTN1
LUZP6
LY6G5B
LY6G5C
LY6G6C
LY6G6D
LY6G6E
LY6G6F
LY6H
LY86
LYRM2
LYSMD1
LYZL1
LZTFL1
LZTR1
M6PR
MAD2L1BP
MAEA
MAF1
MAGEA12
MAGEA3
MALT1
MAN2A2
MANBAL
MAP2K1
MAP2K3
MAP2K5
MAP3K2
MAP3K3
MAP3K6
MAP3K7
MAP4
MAP4K3
MAP4K5
MAP7D3
MAPK1
MAPK1IP1L
MAPKAPK2
MAPKAPK5
MAPRE1
MARCH5
MARCH7
MARK3
MARVELD2

MAS1L
MAT2B
MAX
MB
MBD1
MBD2
MBIP
MBNL1
MBOAT1
MBTPS1
MBTPS2
MCCC1
MCCC2
MCCD1
MCM9
MCOLN1
MCPH1
MCRS1
MDC1
MDFI
MDM2
MDM4
MED1
MED10
MED11
MED14
MED17
MED19
MED20
MED29
MED31
MED6
MEF2A
METTL11B
METTL13
METTL14
METTL18
METTL22
METTL24
METTL25
METTL2B
METTL3
METTL6
METTL9
MEX3C
MFAP1

MFAP3
MFAP3L
MFSD8
MGA
MGAT4B
MGC15885
MGC27382
MGC34034
MGC72080
MGEA5
MGST2
MIB1
MICA
MICB
MIER1
MIR1-1
MIR1-2
MIR100
MIR101-1
MIR103A1
MIR105-1
MIR105-2
MIR106A
MIR107
MIR10A
MIR10B
MIR1178
MIR1179
MIR1180
MIR1181
MIR1182
MIR1185-1
MIR1185-2
MIR1200
MIR1203
MIR1204
MIR1205
MIR1207
MIR1208
MIR122
MIR1224
MIR1225
MIR1226
MIR1228
MIR1229
MIR1234

MIR1237
MIR124-1
MIR124-2
MIR124-3
MIR1243
MIR1246
MIR1247
MIR1249
MIR1250
MIR1251
MIR1252
MIR1253
MIR1256
MIR1257
MIR1258
MIR125A
MIR125B1
MIR125B2
MIR126
MIR1262
MIR1265
MIR1266
MIR127
MIR1272
MIR1275
MIR1276
MIR1278
MIR1279
MIR128-2
MIR1281
MIR1283-1
MIR1283-2
MIR1284
MIR1286
MIR1287
MIR1288
MIR1289-2
MIR129-1
MIR129-2
MIR1292
MIR1296
MIR1297
MIR1301
MIR1304
MIR1305
MIR1306

MIR130A
MIR130B
MIR132
MIR1322
MIR1323
MIR1324
MIR133A1
MIR133B
MIR134
MIR135A1
MIR135A2
MIR135B
MIR136
MIR137
MIR138-1
MIR138-2
MIR140
MIR141
MIR142
MIR143
MIR145
MIR1468
MIR1469
MIR146B
MIR1470
MIR1471
MIR147B
MIR148A
MIR148B
MIR150
MIR152
MIR153-2
MIR1538
MIR1539
MIR154
MIR155
MIR15A
MIR15B
MIR16-1
MIR16-2
MIR181A1
MIR181A2
MIR181B1
MIR181B2
MIR181C
MIR181D

MIR182
MIR1827
MIR183
MIR184
MIR185
MIR187
MIR188
MIR1908
MIR190B
MIR191
MIR1910
MIR1911
MIR1912
MIR1913
MIR1914
MIR1915
MIR192
MIR193A
MIR193B
MIR194-2
MIR195
MIR196A1
MIR196A2
MIR196B
MIR197
MIR1973
MIR1976
MIR198
MIR199A1
MIR199A2
MIR199B
MIR19A
MIR200A
MIR200B
MIR200C
MIR202
MIR204
MIR2052
MIR2053
MIR2054
MIR206
MIR208B
MIR20B
MIR21
MIR211
MIR2110

MIR2113
MIR2114
MIR2116
MIR2117
MIR212
MIR215
MIR216A
MIR216B
MIR218-1
MIR218-2
MIR221
MIR222
MIR223
MIR2276
MIR2278
MIR23A
MIR23B
MIR24-1
MIR24-2
MIR26B
MIR27A
MIR27B
MIR296
MIR298
MIR299
MIR29A
MIR29B1
MIR29B2
MIR300
MIR301A
MIR301B
MIR302A
MIR302D
MIR30A
MIR30B
MIR30C1
MIR30C2
MIR30D
MIR30E
MIR32
MIR320B1
MIR320C1
MIR320C2
MIR320D1
MIR320D2
MIR328

MIR329-1
MIR329-2
MIR330
MIR331
MIR335
MIR337
MIR339
MIR33A
MIR340
MIR345
MIR346
MIR34B
MIR363
MIR365A
MIR367
MIR369
MIR372
MIR373
MIR374A
MIR374B
MIR375
MIR376B
MIR377
MIR379
MIR380
MIR381
MIR382
MIR383
MIR384
MIR409
MIR410
MIR411
MIR412
MIR423
MIR424
MIR425
MIR429
MIR431
MIR432
MIR433
MIR449A
MIR449B
MIR449C
MIR450A1
MIR450B
MIR454

MIR455
MIR483
MIR484
MIR485
MIR487A
MIR487B
MIR488
MIR489
MIR490
MIR493
MIR494
MIR495
MIR496
MIR498
MIR502
MIR503
MIR504
MIR505
MIR506
MIR507
MIR508
MIR509-1
MIR509-2
MIR509-3
MIR510
MIR512-1
MIR512-2
MIR514A3
MIR515-1
MIR515-2
MIR516A1
MIR516A2
MIR516B1
MIR516B2
MIR517A
MIR517B
MIR517C
MIR518A1
MIR518A2
MIR518B
MIR518C
MIR518D
MIR518E
MIR518F
MIR519A1
MIR519A2

MIR519B
MIR519C
MIR519D
MIR519E
MIR520A
MIR520B
MIR520E
MIR520F
MIR520G
MIR521-1
MIR522
MIR523
MIR524
MIR525
MIR526A1
MIR526A2
MIR526B
MIR532
MIR539
MIR542
MIR543
MIR545
MIR548A1
MIR548B
MIR548C
MIR548D2
MIR548F1
MIR548F2
MIR548F3
MIR548F5
MIR548G
MIR548H3
MIR548H4
MIR548I2
MIR548I3
MIR548I4
MIR548J
MIR548N
MIR548Q
MIR551B
MIR553
MIR556
MIR557
MIR558
MIR559
MIR563

MIR567
MIR568
MIR572
MIR573
MIR574
MIR575
MIR577
MIR578
MIR580
MIR581
MIR583
MIR585
MIR590
MIR591
MIR592
MIR595
MIR596
MIR597
MIR598
MIR599
MIR600
MIR601
MIR602
MIR603
MIR604
MIR605
MIR609
MIR611
MIR613
MIR614
MIR617
MIR618
MIR620
MIR621
MIR626
MIR627
MIR628
MIR629
MIR632
MIR636
MIR637
MIR638
MIR639
MIR643
MIR648
MIR651

MIR653
MIR654
MIR655
MIR656
MIR657
MIR661
MIR662
MIR663A
MIR665
MIR668
MIR670
MIR671
MIR675
MIR7-2
MIR7-3
MIR708
MIR711
MIR718
MIR744
MIR758
MIR759
MIR760
MIR761
MIR762
MIR764
MIR765
MIR767
MIR769
MIR770
MIR802
MIR874
MIR877
MIR885
MIR888
MIR889
MIR890
MIR891A
MIR891B
MIR892A
MIR892B
MIR9-1
MIR9-3
MIR920
MIR921
MIR922
MIR92A1

MIR92B
MIR933
MIR938
MIR940
MIR941-1
MIR941-3
MIR942
MIR943
MIR96
MIR98
MIR99A
MIRLET7A1
MIRLET7A2
MIRLET7A3
MIRLET7B
MIRLET7C
MIRLET7D
MIRLET7F2
MIRLET7G
MIRLET7I
MITD1
MKLN1
MKNK1
MKRN2
MLF2
MLLT3
MLN
MLXIPL
MMAA
MMADHC
MMP23A
MMP23B
MMS19
MNAT1
MOB1A
MOCS3
MOG
MORC2
MORC3
MOXD2P
MPHOSPH10
MPHOSPH8
MPND
MPP5
MPP7
MPRIP

MRAP
MRC1
MRFAP1L1
MRI1
MRPL14
MRPL9
MRPS10
MRPS14
MRPS15
MRPS18B
MRPS26
MS4A13
MS4A6E
MSH3
MSH5
MSL1
MSL2
MT1B
MT1E
MT4
MTA2
MTERFD3
MTF1
MTFMT
MTFR1L
MTG1
MTHFD2
MTIF3
MTM1
MTMR10
MTMR12
MTMR14
MTMR2
MTMR4
MTMR6
MTOR
MTRF1
MUC21
MYB
MYBBP1A
MYL10
MYL12A
MYL6B
MYNN
MYO1E
MYSM1

N4BP1
NAA16
NAA25
NAA30
NAA35
NAA60
NACC2
NADK
NADSYN1
NANP
NAPA
NAPB
NAPG
NARF
NARS
NAT10
NBAS
NBN
NCEH1
NCK1
NCKAP1
NCOR1
NCR3
NCRUPAR
NDFIP1
NDFIP2
NDNL2
NDRG3
NDUFA11
NDUFB11
NEDD8
NEK7
NELFA
NELFCD
NELFE
NEMF
NEU1
NF2
NFATC2IP
NFATC3
NFKBID
NFRKB
NHEG1
NIN
NIPA2
NKIRAS1

NKIRAS2
NKTR
NKX6-3
NLK
NLRX1
NME2
NME6
NMNAT1
NMS
NMT1
NMT2
NOL3
NONO
NOTCH2
NOTCH4
NPEPPS
NPHP3
NPRL3
NPSR1-AS1
NPTN
NPY
NR2C1
NRAS
NRBP2
NRD1
NRDE2
NRM
NSD1
NSFL1C
NSMCE2
NSRP1
NSUN4
NT5C3B
NUB1
NUBP1
NUDT12
NUDT13
NUDT16L1
NUDT18
NUDT9
NUP153
NUP160
NUP214
NUP98
NUPL1
NXF1

NXF5
OCIAD1
OCM
OCM2
OCRL
OFD1
OGDHL
OGFOD3
OPN1LW
OPN1MW
OPN1MW2
OR10C1
OR11A1
OR12D2
OR14J1
OR2B3
OR2H1
OR2H2
OR2J2
OR2T12
OR2T29
OR2T33
OR2T5
OR2T8
OR2W1
OR4F5
OR5V1
OR7E37P
ORAI1
ORC4
ORMDL2
ORMDL3
OSBPL10
OSBPL3
OSTF1
OTUB1
OTUD4
OXA1L
OXLD1
OXT
P2RX6P
PABPN1L
PACS2
PAF1
PAFAH1B2
PAGE3

PAIP2B
PAK2
PAK4
PALB2
PAN2
PANK2
PANK3
PAOX
PAPD4
PAPD7
PAPOLA
PAPSS1
PARG
PARN
PARP11
PARP6
PATL1
PAX9
PAXBP1
PAXIP1
PBDC1
PCBP1
PCF11
PCMTD2
PCNAP1
PCNX
PCSK7
PCYOX1
PDCD10
PDCD6
PDCD6IP
PDCD7
PDHB
PDLIM5
PDPK1
PDPR
PDS5A
PDS5B
PDSS2
PDXDC1
PELP1
PEMT
PER4
PEX1
PEX10
PEX11B

PEX12
PEX19
PEX2
PEX26
PEX5
PFDN5
PFDN6
PFN1
PGA3
PGBD2
PGGT1B
PGK1
PHACTR4
PHAX
PHC3
PHF1
PHF14
PHF20
PHF20L1
PHF23
PHF6
PHGR1
PHKA1
PHKB
PHLPP1
PHLPP2
PHTF2
PI4KA
PI4KB
PICALM
PIGB
PIGC
PIGL
PIGO
PIGP
PIGS
PIGT
PIH1D1
PIH1D3
PIK3R4
PIP4K2B
PISRT1
PITPNB
PKM
PKNOX1
PLA2G2C

PLA2G2E
PLAA
PLAGL2
PLEKHA1
PLEKHA3
PLEKHA8P1
PLEKHF2
PLEKHG3
PLRG1
PLS3
PMM2
PMP2
PMPCB
PMS2
PNKP
PNPLA2
PNPLA6
POC1B
POC5
POFUT1
POGK
POLI
POLK
POLL
POLR2A
POLR2B
POLR2C
POLR2E
POLR3A
POLR3C
POLR3E
POM121
POM121C
POMT1
POT1
POTED
PPA2
PPBPP2
PPHLN1
PPIC
PPIE
PPIL2
PPIL4
PPIP5K1
PPIP5K2
PPM1A

PPM1D
PPP1CB
PPP1R10
PPP1R12A
PPP1R12B
PPP1R13L
PPP1R15B
PPP1R18
PPP1R2
PPP1R35
PPP1R3D
PPP1R42
PPP2CA
PPP2CB
PPP2R1A
PPP2R2A
PPP2R2D
PPP2R5A
PPP2R5C
PPP2R5E
PPP4R1
PPP4R2
PPP6C
PPP6R3
PPT2
PPTC7
PPWD1
PQLC1
PRAF2
PRAMEF2
PRAMEF20
PRCC
PRDM15
PRDM2
PRDX4
PRDX5
PREP
PRKACA
PRKAG2
PRKCI
PRKCSH
PRLH
PRMT2
PRMT6
PRNP
PROSER1

PRPF18
PRPF3
PRPF31
PRPF38B
PRPF40A
PRPF8
PRPSAP1
PRPSAP2
PRR15L
PRR21
PRR3
PRR5
PRRT1
PRSS41
PRSS54
PSENEN
PSMA1
PSMA3
PSMA4
PSMA5
PSMA8
PSMB2
PSMB3
PSMB4
PSMB5
PSMB6
PSMB8
PSMB9
PSMC2
PSMC4
PSMC5
PSMC6
PSMD11
PSMD12
PSMD3
PSMD5
PSMD9
PSME4
PSORS1C1
PSORS1C2
PSORS1C3
PSPC1
PTAR1
PTBP1
PTDSS2
PTGR2

PTH2
PTPN1
PTPN11
PTPN2
PTPRA
PUM2
PURB
PUS7L
PUSL1
PVR
PVRL4
PWRN1
PYCR2
PYGO2
PYHIN1
QARS
QRICH1
RAB10
RAB11A
RAB11FIP2
RAB18
RAB1B
RAB21
RAB22A
RAB24
RAB2A
RAB35
RAB3D
RAB3GAP1
RAB3GAP2
RAB5A
RAB6A
RAB7A
RABEP1
RABGAP1
RABGGTA
RABIF
RAC1
RAD21
RAD23B
RAD50
RAD52
RAD9A
RAE1
RAF1
RALA

RALBP1
RALGAPB
RANBP2
RANBP6
RANBP9
RAP1A
RAP1B
RAP2A
RAP2B
RARS
RASA3
RASEF
RBAK
RBBP5
RBBP6
RBFOX2
RBM15
RBM15B
RBM18
RBM22
RBM25
RBM26
RBM27
RBM33
RBM38
RBM39
RBM4
RBM45
RBM5
RBM6
RBMXL1
RBPJ
RCAN3
RCC2
RCN2
RCOR1
RDH14
RDX
REG1B
RELL2
REPS1
RER1
RERE
RETNLB
REV1
REV3L

REXO2
RFC1
RFK
RFWD2
RFXANK
RGL2
RGMB
RGPD5
RGS13
RGS3
RHBDD1
RHOA
RHOG
RHOT1
RIC8A
RING1
RINT1
RIOK2
RLF
RLIM
RMDN3
RMND5A
RMND5B
RNASE9
RNASEH1
RNASEK
RNF11
RNF111
RNF115
RNF121
RNF13
RNF130
RNF14
RNF144B
RNF168
RNF170
RNF185
RNF187
RNF19B
RNF20
RNF216P1
RNF220
RNF38
RNF39
RNF4
RNF5

RNF5P1
RNF6
RNF8
RNPEP
RNU12
RNU5E-1
RNY5
RPAIN
RPAP1
RPAP2
RPAP3
RPE
RPL13AP17
RPL13AP5
RPL19
RPL23AP32
RPL29P2
RPL3
RPL36A
RPL36AL
RPL8
RPP14
RPP21
RPP38
RPRD2
RPS15
RPS18
RPS4X
RPS4Y2
RPS5
RPS6KA5
RPS6KB1
RPS6KC1
RPS9
RPUSD2
RPUSD3
RRAS
RRM2B
RRNAD1
RSRC2
RTCA
RTF1
RTTN
RUFY1
RUFY2
RXRB

RYBP
S100A7L2
S100G
S100PBP
S100Z
SAA3P
SACM1L
SAFB
SAFB2
SAMD1
SAP130
SAP30BP
SAP30L
SAPCD1
SAR1A
SARS
SARS2
SART1
SART3
SBF2
SCAF11
SCAF4
SCAF8
SCAMP1
SCAMP3
SCAND2P
SCAND3
SCARB2
SCARNA1
SCARNA14
SCARNA18
SCARNA20
SCARNA23
SCFD1
SCGB1D4
SCNM1
SCP2
SCRN1
SCRN2
SCYL2
SDAD1
SDC4
SDC4P
SDCCAG3
SDE2
SDHAF1

SDHAF2
SDR42E1
SEC11A
SEC23IP
SEC24B
SEC61A2
SEC62
SEMA4F
SENP1
SENP5
SENP6
SENP7
SEPN1
SEPSECS
SERF2
SETD2
SETD3
SETD4
SETD8
SETDB1
SETDB2
SF3A1
SF3B1
SF3B3
SF3B4
SFSWAP
SFT2D1
SFT2D3
SFTA1P
SFTA2
SFTA3
SFXN1
SGPL1
SGSM1
SH2B1
SH2D4A
SH3GLB1
SH3YL1
SHARPIN
SHMT2
SHOC2
SHPK
SIKE1
SIPA1L1
SIRT1
SIRT3

SKAP2
SKI
SKIV2L
SKIV2L2
SKP1
SLAIN1
SLC12A6
SLC12A8
SLC15A4
SLC19A2
SLC20A2
SLC25A14
SLC25A16
SLC25A36
SLC25A38
SLC25A3P1
SLC25A44
SLC25A46
SLC30A9
SLC35A1
SLC35A3
SLC35B4
SLC35D1
SLC35F2
SLC35G1
SLC36A4
SLC38A6
SLC39A1
SLC39A11
SLC39A7
SLC39A9
SLC41A1
SLC44A4
SLC4A1AP
SLC4A2
SLC5A3
SLC5A6
SLC7A1
SLC9A6
SLC9A7
SLMAP
SLTM
SLX1A
SLX1B
SMAD1
SMAD2

SMAD5
SMAP1
SMARCA4
SMARCA5
SMARCAD1
SMARCAL1
SMARCB1
SMARCC1
SMARCC2
SMARCD1
SMARCD2
SMARCE1
SMEK1
SMEK2
SMG1
SMG5
SMG6
SMG7
SMG9
SMIM15
SMIM7
SMPD2
SMPDL3A
SMU1
SMYD4
SNAP47
SNAPC1
SNAPC3
SNAPIN
SNAR-C3
SNAR-D
SNAR-E
SNAR-F
SNAR-G1
SNAR-G2
SNAR-H
SNAR-I
SNHG7
SNORA11B
SNORA13
SNORA26
SNORA30
SNORA35
SNORA36C
SNORA37
SNORA38

SNORA49
SNORA54
SNORA55
SNORA59A
SNORA74B
SNORA79
SNORA7A
SNORA80B
SNORD100
SNORD103A
SNORD105
SNORD107
SNORD109A
SNORD11
SNORD110
SNORD111
SNORD111B
SNORD113-1
SNORD113-2
SNORD113-4
SNORD113-5
SNORD113-6
SNORD113-7
SNORD113-9
SNORD114-1
SNORD114-10
SNORD114-11
SNORD114-12
SNORD114-13
SNORD114-14
SNORD114-15
SNORD114-16
SNORD114-17
SNORD114-18
SNORD114-19
SNORD114-20
SNORD114-21
SNORD114-22
SNORD114-23
SNORD114-25
SNORD114-26
SNORD114-27
SNORD114-28
SNORD114-29
SNORD114-3
SNORD114-30

SNORD114-31
SNORD114-4
SNORD114-5
SNORD114-6
SNORD114-7
SNORD114-8
SNORD114-9
SNORD115-1
SNORD115-10
SNORD115-11
SNORD115-12
SNORD115-13
SNORD115-14
SNORD115-15
SNORD115-16
SNORD115-2
SNORD115-21
SNORD115-22
SNORD115-24
SNORD115-25
SNORD115-27
SNORD115-28
SNORD115-3
SNORD115-30
SNORD115-31
SNORD115-32
SNORD115-33
SNORD115-35
SNORD115-37
SNORD115-38
SNORD115-39
SNORD115-4
SNORD115-40
SNORD115-41
SNORD115-44
SNORD115-48
SNORD115-5
SNORD115-6
SNORD115-7
SNORD115-8
SNORD115-9
SNORD116-1
SNORD116-10
SNORD116-11
SNORD116-12
SNORD116-13

SNORD116-14
SNORD116-15
SNORD116-16
SNORD116-18
SNORD116-19
SNORD116-2
SNORD116-20
SNORD116-21
SNORD116-22
SNORD116-23
SNORD116-24
SNORD116-25
SNORD116-26
SNORD116-28
SNORD116-29
SNORD116-4
SNORD116-7
SNORD116-8
SNORD116-9
SNORD119
SNORD123
SNORD124
SNORD125
SNORD126
SNORD127
SNORD18A
SNORD18C
SNORD1A
SNORD1B
SNORD1C
SNORD2
SNORD24
SNORD26
SNORD27
SNORD29
SNORD30
SNORD32B
SNORD34
SNORD36A
SNORD36B
SNORD36C
SNORD37
SNORD38A
SNORD38B
SNORD41
SNORD42A

SNORD42B
SNORD43
SNORD45C
SNORD48
SNORD4A
SNORD50A
SNORD50B
SNORD53
SNORD54
SNORD55
SNORD56
SNORD56B
SNORD57
SNORD58A
SNORD59A
SNORD59B
SNORD6
SNORD63
SNORD66
SNORD7
SNORD71
SNORD72
SNORD74
SNORD75
SNORD78
SNORD8
SNORD80
SNORD81
SNORD82
SNORD84
SNORD85
SNORD86
SNORD88A
SNORD91B
SNORD92
SNORD93
SNORD95
SNORD96A
SNORD98
SNRNP27
SNRNP35
SNRNP48
SNTB2
SNTG1
SNUPN
SNW1

SNX12
SNX14
SNX15
SNX17
SNX2
SNX4
SNX6
SOCS5
SOCS7
SOD1
SON
SP1
SP2
SP3
SPACA5
SPAG7
SPAG9
SPANXA2
SPANXC
SPANXN1
SPANXN4
SPAST
SPATA19
SPATA31A5
SPATA31A6
SPATS1
SPECC1L
SPG11
SPG7
SPHAR
SPICE1
SPIN1
SPIN3
SPINK9
SPNS1
SPOP
SPPL3
SPRR2B
SPRR2C
SPRR2E
SPRR2G
SPRR4
SPRYD3
SPSB3
SPTLC1
SPTLC2

SRA1
SRF
SRFBP1
SRP14
SRP72
SRPK1
SRPK2
SRPRB
SRRD
SRRM1
SRSF4
SRSF8
SRSF9
SS18
SS18L1
SS18L2
SSH1
SSU72
SSX7
ST7-AS2
ST7L
STAG1
STAG3L1
STAM
STAM2
STAMBP
STAP1
STARD3
STAT5B
STATH
STAU1
STH
STK11
STK16
STK19
STK25
STRADA
STRAP
STRBP
STX17
STX7
STXBP3
STXBP5
STYX
SUCLG2
SUDS3

SUGP1
SUGP2
SULT6B1
SUMF2
SUMO1P1
SUMO3
SUN1
SUN5
SUPT20H
SUPT4H1
SUPT5H
SUPT7L
SYNM
SYS1
SZRD1
TACC1
TADA2A
TAF1
TAF1C
TAF2
TAF3
TAF4
TAF6L
TAF7
TAGLN2
TANGO2
TAP1
TARBP2
TASP1
TBC1D12
TBC1D14
TBC1D15
TBC1D21
TBC1D22B
TBC1D28
TBC1D3P2
TBC1D8
TBCC
TBCK
TBL1XR1
TBL2
TBX18
TBX3
TCEB2
TCEB3
TCF20

TCF25
TCP10
TDG
TDP2
TDRD3
TEN1
TERF1
TERF2
TEX10
TEX26
TEX261
TEX28
TFAP2A
TFAP2C
TFCP2
TFIP11
TFPT
THAP1
THOC2
THRAP3
THUMPD1
THUMPD3
THYN1
TIA1
TIAL1
TIAM1
TIGD6
TIMMDC1
TINAGL1
TIPRL
TJP1
TK1
TK2
TLK1
TLK2
TM2D1
TM2D2
TM2D3
TM9SF4
TMBIM4
TMBIM6
TMCO1
TMCO4
TMED10P1
TMED4
TMEM102

TMEM106B
TMEM11
TMEM110
TMEM115
TMEM123
TMEM135
TMEM161B
TMEM164
TMEM167A
TMEM167B
TMEM168
TMEM181
TMEM186
TMEM192
TMEM205
TMEM222
TMEM230
TMEM244
TMEM245
TMEM256
TMEM30C
TMEM33
TMEM39B
TMEM41A
TMEM43
TMEM50A
TMEM55A
TMEM60
TMEM62
TMEM65
TMEM68
TMEM86B
TMEM88B
TMPRSS11BNL
TMSB4Y
TMX1
TMX3
TNF
TNFRSF10A
TNPO1
TNPO3
TNRC6A
TNRC6C
TNXB
TOB2
TOM1L1

TOP2B
TOPORS
TOR1A
TOR1AIP1
TOR1AIP2
TOR1B
TOR3A
TOX4
TP53
TP53BP2
TP53RK
TPD52
TPGS2
TPI1
TPM4
TRA2A
TRAF3IP1
TRAF7
TRAPPC10
TRAPPC11
TRAPPC2P1
TRAPPC3
TRAPPC6B
TRAPPC8
TRAPPC9
TRIL
TRIM10
TRIM15
TRIM2
TRIM23
TRIM24
TRIM25
TRIM26
TRIM27
TRIM31
TRIM33
TRIM34
TRIM39
TRIM4
TRIM40
TRIM52
TRMT1
TRMT10B
TRMT61B
TRNT1
TRPM7

TRPT1
TRUB2
TSC2
TSEN34
TSG101
TSN
TSNAX
TSPO
TSPY2
TSPY4
TSSK2
TTC14
TTC23
TTC31
TTC37
TTC38
TTC39A
TTC7A
TTC8
TTF1
TTI1
TTLL11
TTLL4
TTPAL
TTTY13
TTTY14
TTTY18
TTTY19
TUBB
TUBG2
TUSC2
TUT1
TWSG1
TXLNA
TXNDC15
TXNDC9
TXNL1
TXNL4B
TYW1
TYW5
U2AF2
UBA1
UBA3
UBA5
UBA52
UBAP2

UBD
UBE2A
UBE2B
UBE2D3
UBE2F
UBE2G1
UBE2J2
UBE2K
UBE2L3
UBE2Q1
UBE2R2
UBE2V1
UBE2Z
UBE3A
UBE3C
UBE4A
UBL5
UBLCP1
UBN1
UBOX5
UBQLN1
UBR2
UBR3
UBXN2A
UBXN2B
UCHL3
UCK1
UFC1
UFL1
UGGT2
UGT2A3
UGT2B15
UHMK1
UIMC1
ULK4P3
UNC119B
UNC50
UNK
UPF2
UQCR11
URB1
UROS
USE1
USF2
USP10
USP12

USP14
USP15
USP16
USP21
USP25
USP3
USP37
USP39
USP42
USP47
USP7
UTP23
UTP3
UTS2
UVRAG
VAMP3
VAPB
VARS
VARS2
VAV1
VENTXP1
VENTXP7
VHL
VIP
VIPAS39
VMP1
VPRBP
VPS11
VPS13A
VPS16
VPS26A
VPS28
VPS33A
VPS33B
VPS35
VPS36
VPS37C
VPS41
VPS45
VPS4B
VPS52
VPS72
VPS8
VRK2
VSTM1
VTRNA1-2

VWA7
VWA9
WAC
WAPAL
WARS2
WASF2
WBSCR16
WDFY1
WDR1
WDR11
WDR19
WDR20
WDR26
WDR33
WDR37
WDR45B
WDR48
WDR55
WDR59
WDR70
WDR73
WDR82
WDR83OS
WDR89
WDR92
WDYHV1
WFDC11
WFDC6
WHAMM
WIBG
WIZ
WNK1
WRAP73
WTAP
XAB2
XPC
XPNPEP1
XPO7
XPOT
YAF2
YDJC
YIPF4
YKT6
YLPM1
YME1L1
YTHDC1

YTHDC2
YTHDF1
YTHDF3
YWHAB
YWHAG
YY1
YY1AP1
ZBED4
ZBED5
ZBTB11
ZBTB12
ZBTB14
ZBTB22
ZBTB25
ZBTB3
ZBTB34
ZBTB40
ZBTB43
ZBTB44
ZBTB45
ZBTB48
ZBTB5
ZBTB6
ZBTB8A
ZBTB9
ZC3H11A
ZC3H13
ZC3H14
ZC3H18
ZC3H7A
ZCCHC10
ZCCHC3
ZCCHC4
ZCCHC7
ZCCHC8
ZDHHC17
ZDHHC20
ZFAND1
ZFP1
ZFP3
ZFP57
ZFP91
ZFR
ZFX
ZFYVE20
ZHX3

ZKSCAN2
ZKSCAN4
ZMAT2
ZMPSTE24
ZMYM1
ZMYM5
ZMYM6
ZMYND11
ZNF107
ZNF12
ZNF131
ZNF134
ZNF138
ZNF140
ZNF146
ZNF148
ZNF17
ZNF174
ZNF18
ZNF180
ZNF2
ZNF202
ZNF207
ZNF227
ZNF23
ZNF230
ZNF234
ZNF24
ZNF252P
ZNF26
ZNF260
ZNF268
ZNF271
ZNF302
ZNF304
ZNF311
ZNF317
ZNF318
ZNF320
ZNF321P
ZNF326
ZNF337
ZNF35
ZNF350
ZNF354A
ZNF354B

ZNF394
ZNF398
ZNF410
ZNF416
ZNF419
ZNF420
ZNF428
ZNF433
ZNF44
ZNF440
ZNF445
ZNF449
ZNF45
ZNF451
ZNF461
ZNF468
ZNF510
ZNF512
ZNF512B
ZNF518A
ZNF529
ZNF547
ZNF548
ZNF557
ZNF561
ZNF562
ZNF57
ZNF586
ZNF594
ZNF595
ZNF597
ZNF600
ZNF613
ZNF619
ZNF621
ZNF622
ZNF624
ZNF630
ZNF639
ZNF644
ZNF664
ZNF669
ZNF672
ZNF691
ZNF692
ZNF696

ZNF7
ZNF700
ZNF705G
ZNF75A
ZNF76
ZNF761
ZNF766
ZNF780A
ZNF780B
ZNF782
ZNF783
ZNF787
ZNF789
ZNF800
ZNF805
ZNF830
ZNF839
ZNF92
ZNHIT3
ZNRD1
ZNRF3
ZRANB1
ZRANB2
ZSCAN16
ZSCAN20
ZSCAN22
ZSCAN25
ZSCAN26
ZSCAN29
ZSCAN32
ZSCAN5A
ZSCAN9
ZSWIM5
ZSWIM7
ZW10
ZXDC
ZZEF1
ZZZ3
